# Pathways of aging: comparative analysis of gene signatures in replicative senescence and stress induced premature senescence

**DOI:** 10.1186/s12864-016-3352-4

**Published:** 2016-12-28

**Authors:** Kamil C. Kural, Neetu Tandon, Mikhail Skoblov, Olga V. Kel-Margoulis, Ancha V. Baranova

**Affiliations:** 1 School of Systems Biology, George Mason University, Manassas, VA 20110 USA; 2 GeneXplain GmbH, 38302 Wolfenbüttel, Germany; 3 Research Centre for Medical Genetics, Moscow, Russia; 4 Moscow Institute of Physics and Technology, Dolgoprudny, 141700 Russia

## Abstract

**Background:**

In culturing normal diploid cells, senescence may either happen naturally, in the form of replicative senescence, or it may be a consequence of external challenges such as oxidative stress. Here we present a comparative analysis aimed at reconstruction of molecular cascades specific for replicative (RS) and stressinduced senescence (SIPS) in human fibroblasts.

**Results:**

An involvement of caspase-3/keratin-18 pathway and serine/threonine kinase Aurora A/ MDM2 pathway was shared between RS and SIPS. Moreover, stromelysin/MMP3 and N-acetylglucosaminyltransferase enzyme MGAT1, which initiates the synthesis of hybrid and complex Nglycans, were identified as key orchestrating components in RS and SIPS, respectively. In RS only, Aurora-B driven cell cycle signaling was deregulated in concert with the suppression of anabolic branches of the fatty acids and estrogen metabolism. In SIPS, Aurora-B signaling is deprioritized, and the synthetic branches of cholesterol metabolism are upregulated, rather than downregulated. Moreover, in SIPS, proteasome/ubiquitin ligase pathways of protein degradation dominate the regulatory landscape. This picture indicates that SIPS proceeds in cells that are actively fighting stress which facilitates premature senescence while failing to completely activate the orderly program of RS. The promoters of genes differentially expressed in either RS or SIPS are unusually enriched by the binding sites for homeobox family proteins, with particular emphasis on HMX1, IRX2, HDX and HOXC13. Additionally, we identified Iroquois Homeobox 2 (IRX2) as a master regulator for the secretion of SPP1-encoded osteopontin, a stromal driver for tumor growth that is overexpressed by both RS and SIPS fibroblasts. The latter supports the hypothesis that senescence-specific de-repression of *SPP1* aids in SIPS-dependent stromal activation.

**Conclusions:**

Reanalysis of previously published experimental data is cost-effective approach for extraction of additional insignts into the functioning of biological systems.

**Electronic supplementary material:**

The online version of this article (doi:10.1186/s12864-016-3352-4) contains supplementary material, which is available to authorized users.

## Background

All biological organisms share a universal feature called aging. In multicellular organisms, the major consequence of aging is a functional deficiency of cells, tissues and organs. Additionally, renewable cells and tissues display deficits in regenerative capacities that are paralleled by an increase in incidence of hyperplasia, a gain-of-functional change that allow cells to proliferate inappropriately [1]. The most serious type of hyperplasia is known as cancer.

In order to understand the aging process, model experiments are of crucial importance. Majority of well-known cellular models were developed at the time of the boom in cell and tissue culturing, providing a trove of important insights into cellular physiology. In particular, one of the pioneers in cell culture, Leonard Hayflick, showed that normal, non-transformed cells in culture can go through a limited number of divisions upon reaching the end of their replicative life span [2]. This finite number of divisions has been termed as the Hayflick limit.

Over the decades, it was discovered that proliferating cells reach the Hayflick limit largely because repeated DNA replication in the absence of telomerase causes telomeres to shorten and eventually affect chromosomal stability and genome functioning [3]. The cells undergoing replicative senescence (RS) became enlarged in size and demonstrate systemic changes in expression level of many genes. The entry into the senescent state is mediated by at least two distinct signaling cascades linked to the activation of two tumor suppressing proteins, the p53/ p21 and p16INK4a/pRB pathways [4]. On the other side, cells exposed to various concentrations of different DNA damaging agents such as bleomycin, tert-butylhydroperoxide, hydrogen peroxide or doses of UV A and UV B also become post-mitotic and display signs of senescence. Latter phenomenon is termed as stress induced premature senescence (SIPS) [5]. The expression levels of many genes are changed during SIPS. It is believed that cellular and molecular mechanisms promoting an entry into senescence also provide protection against tumor formation [6, 7]. Identification and understanding the differences between RS and SIPS senescence is critical for the development of anti-aging strategies that do not induce tumorigenesis.

The main purpose behind this study was to identify the differentially expressed genes DEGs) that distinguish the processes of replicative and stress induced senescence and to reconstruct relevant molecular cascades. To this end, we employed bioinformatics software platform GeneXplain that allowed both upstream and downstream analysis of DEGs validated by three-way comparisons of each type of senescent cells against the young cells (control group) and against each other. In both types of senescence, master regulators genes were identified. We also identified Iroquois Homeobox 2 (IRX2) as the master regulators for an expression of *SPP1*-encoded osteopontin, a secreted stromal driver for tumor growth that is overexpressed by both RS and SIPS fibroblasts.

## Methods

### Microarray data, differential expression analysis

To investigate both types of senescence, publicly available dataset GSE13330 was downloaded from Gene Expression Omnibus (NCBI, Bethesda, MD, USA). This dataset is comprised of 16 samples profiled using Affymetrix Human Genome U133 Plus 2.0 Array. In this dataset, replicative-senescent human foreskin BJ fibroblasts and young fibroblast controls were assayed in 6 biological replicates each. An induction of cell senescence by stress was performed with 100ug/ml of bleomycin sulfate, and analyzed in four biological replicates [8].

Raw data of stress induced and replicative senescence as well as data on younger control cells were normalized and background corrected using RMA (Robust Multi-Array Average). The Limma (Linear Models for Microarray Data) method [9, 10] was applied to define fold changes of genes and to calculate adjusted *p*-values using a Benjamini-Hochberg adjusted *p*-value cutoff (.05). The up regulated genes were filtered using the filter: logFC > 0.5 && adj_P_Val < 0.05. Down regulated genes were filtered using the filter: logFC < −0.5 && adj_P_Val < 0.05.

### Functional enrichment analysis

DEGs were analyzed using geneXplain bioinformatics software platform (http://www.genexplain.com). Using the workflows in geneXplain framework, the sets of up and down regulated genes for both SIPS and RS were mapped to various gene ontologies, i.e. biological processes, cellular components, molecular functions, reactome pathways, TRANSPATH® [11] pathways and transcription factor classification.

The output links each gene to GO identifiers that are, in turn, are hyperlinked to the page http://www.ebi.ac.uk/QuickGO with information about this ontological term. Ontological classification evaluates statistical significance for each term; the resultant *p*-values were used for further interpretation of the results.

### Promoter analysis

The sets of up- and down-regulated genes identified in each comparison were subjected to the promoter analysis using TRANSFAC [12] database of position weight matrices (PWMs) characteristic for vertebrate genomes (vertebrate_non_redundant_minSUM database subdivision). Each promoter was defined as the sequence within −1000 to +100 coordinates, where the TSS of the main transcript of each gene was the point 0.

The TFBS search on promoter sequences was done using the MATCH algorithm [13, 14] integrated in the GeneXplain platform and executed within the pre-defined workflows. The promoter sequences and annotations of TSS positions were accordinh to the Ensembl database (version hg19 build 72.37).

### Identification of master regulators

Lists of DEGs upregulated in each of cell senescence types were used as inputs in a search for master regulatory key molecules that influence the senescence pathways [13]. The search was performed in the TRANSPATH® database networks with a maximum radius of 10 steps upstream of an input gene set, a default cut-off score at 0.2, and for FDR at 0.05 and Z-score at 1.0.

### Pathway studio -guided analysis of ospeopontin regulation

To construct a concise network that bridges senescence regulators highlighted by GeneXPlain–guided analysis of DEGs, we used the Pathway Studio software (Elsevier, Rockville, MD) that is able to dynamically create and draw protein interaction networks and pathways. Each node represents either a molecular entity or a control mechanism of the interaction. In this study, we the shortest path analysis function was utilized predominantly.

## Results

Extraction of gene signatures important in replicative and stress-induced cell senescence was performed using public 16-sample dataset GSE13330 previously described in [8]. We divided the study in two parts. First, we analyzed signaling events that are shared in both RS and SIPS. Second, we identified DEGs and respective signaling events uniquely describing each type of senescence.

**Fig. 1:**
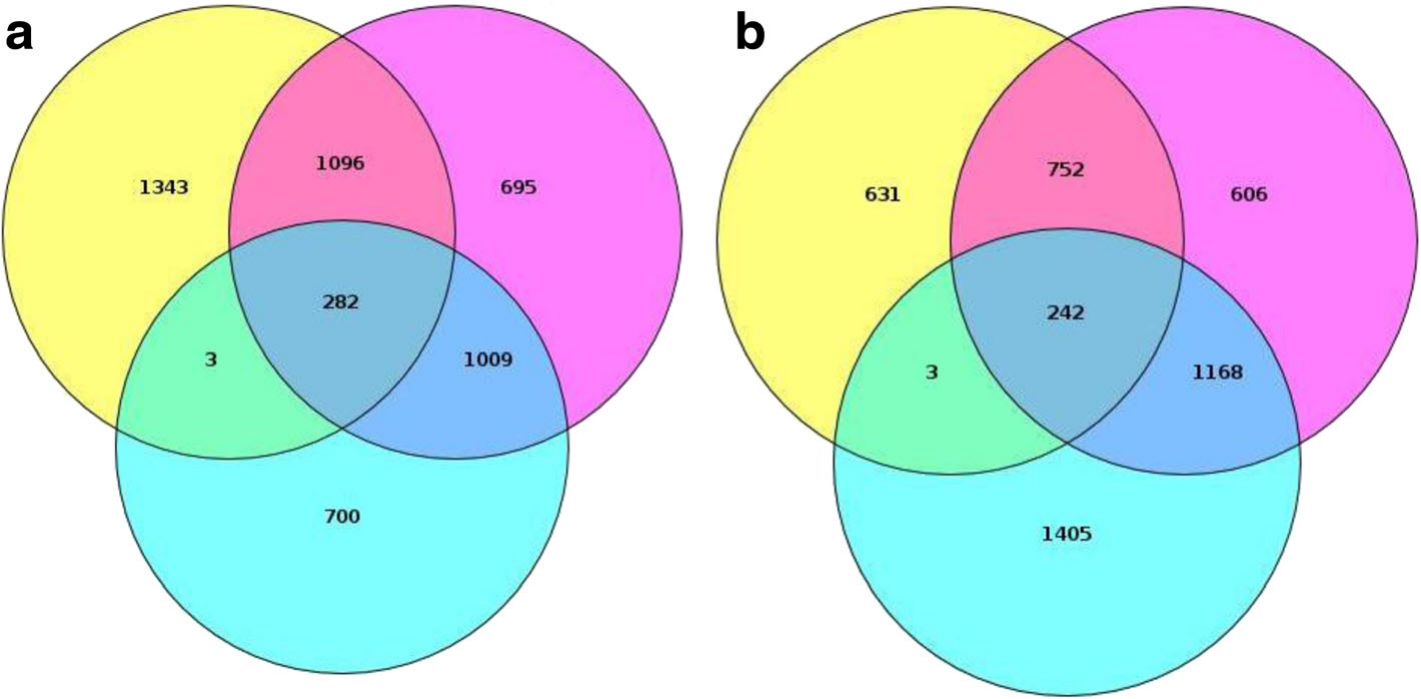
Venn diagrams depicting lists of downregulated (**a**) and upregulated (**b**) genes common and specific for each type of cell senescence. *Yellow circle* represents the comparison of Bleomycin Treated cells to Replicative Senescent cells. *Purple circle* represents the comparison of Bleomycin Treated cells to Young Controls. *Blue circle* represents the comparison of Replicative Senescent cells and Young Controls

To dissect the differences between RS and SIPS, 1) six biological replicates of replicative senescent fibroblasts were compared to six biological replicates of young fibroblasts and yielded 1994 downregulated and 2818 upregulated mRNAs; 2) four biological replicates of bleomycin induced senescent fibroblasts were compared to six replicates of young fibroblast cultures (3082 downregulated and 2768 upregulated mRNAs); 3) six biological replicates of replicative senescent fibroblasts were compared to four biological replicates of bleomycin induced senescent fibroblasts (2724 downregulated and 1628 upregulated mRNAs). Each list of DEGs was divided into up- and downregulated sections. A comparison of the three DEG lists that resulted from comparisons described above have identified 524 shared between RS and SIPS (Fig. 1a and b for downregulated (*N* = 248) and upregulated (*N* = 242) genes, respectively). All these mRNAs exhibited a change in expression levels of more than two fold in all three types of the profiled cells.

### Genes commonly involved in both bleomycin induced and replicative senescence

A total of 1410 genes were upregulated and a total of 1291 genes were downregulated both in RS and SIPS as compared to younger control fibroblasts. Resultant lists of up- and downregulated genes were subjected to functional analysis separately. Each gene was mapped to GO biological processes, GO cellular components, GO molecular functions, Reactome, HumanCyc, TF classification and the latest TRANSPATH® [11] available in the geneXplain platform.

Caspase-3/keratin-18 and Aurora A kinase/MDM2 pathways were the most upregulated signaling events commonly dominating regulatory landscapes in both bleomycin-induced and replicative type of senescence (adjusted *P*-values < 0.009 for each of these signaling events). Concerted upregulation of many enzymes participating in glutamate (ABAT, GCLM, GLS), nucleotide (PNP, NT5E, NAMPT, NMNAT2, AMPD3), polyamine (ABT, ODC1) and choline (EPT1, PLCB4) metabolic branches was also noted (adjusted *p*-value range of <0.016 to < 0.05 for various fragments of these metabolic cascades) (Table 1).

**Table 1:** Results of pathway fragment analysis

Pathway fragment analysis						
Pathway fragments down-regulated in both RS and SIPS						
Title	Number of hits	Group size	Expected hits	*P*-value	Adjusted *P*-value	Hit names
Glu ---GluR1:GluR3--- > c-fos	2	11	0.11067	0.00504	0.02521	GRIA1 (ENSG00000155511), GRIA1 (ENSG00000269977)
GluR1:GluR2 complex	2	9	0.09055	0.00334	0.02521	GRIA1 (ENSG00000155511), GRIA1 (ENSG00000269977)
AMPA receptor signaling	2	14	0.14086	0.00819	0.0273	GRIA1 (ENSG00000155511), GRIA1 (ENSG00000269977)
wnt --- > beta-catenin	2	25	0.25153	0.02525	0.04864	TCF7L2, WNT2
SDF-1 --- > G-protein	2	27	0.27165	0.02918	0.04864	CXCL12, GNG2
SDF-1 --- > calcium mobilization	2	26	0.26159	0.02718	0.04864	CXCL12, GNG2
Pathway fragments up-regulated in both RS and SIPS						
Caspase-3 ---/ K18	2	3	0.04856	7.5719E-4	0.00909	CASP3, KRT18
Aurora-A(h) ---/ p53(h)	2	3	0.04856	7.5719E-4	0.00909	AURKA, MDM2
glutamate metabolism	3	16	0.25897	0.00189	0.01513	ABAT, GCLM, GLS
L-glutamate ---ammonia--- > 2-oxoglutarate	2	8	0.12948	0.00671	0.01611	ABAT, GLS
xanthosine-5-phosphate --- > allantoin	2	8	0.12948	0.00671	0.01611	NT5E, PNP
IMP --- > xanthine	2	8	0.12948	0.00671	0.01611	NT5E, PNP
dGDP --- > guanine	2	7	0.1133	0.00509	0.01611	NT5E, PNP
dADP --- > hypoxanthine	2	7	0.1133	0.00509	0.01611	NT5E, PNP
L-ornithine --- > succinate	2	8	0.12948	0.00671	0.01611	ABAT, ODC1
polyamine metabolism	2	8	0.12948	0.00671	0.01611	ABAT, ODC1
plasmenylethanolamine --- > plasmenylcholine	2	10	0.16185	0.01057	0.02307	EPT1, PLCB4
GDP --- > xanthine	2	12	0.19423	0.01519	0.03039	NT5E, PNP
interconversions and degradations of purine ribonucleotides	3	41	0.6636	0.02728	0.04365	AMPD3, NT5E, PNP
L-tryptophan --- > NAD+, NADPH	2	16	0.25897	0.02653	0.04365	NAMPT, NMNAT2
biosynthesis and degradation of nicotinamide,NAD+,NADP+	2	16	0.25897	0.02653	0.04365	NAMPT, NMNAT2
plasmenylcholine biosynthesis	2	19	0.30752	0.03667	0.05501	EPT1, PLCB4
Pathway fragments down-regulated in RS						
acetyl-CoA, acetoacetyl-CoA --- > cholesterol, fatty acid	7	21	0.90945	1.6325E-5	5.7683E-4	FDFT1, FDPS, HMGCS1, IDI1, LSS, MVD, SQLE
cholesterol metabolism	7	21	0.90945	1.6325E-5	5.7683E-4	FDFT1, FDPS, HMGCS1, IDI1, LSS, MVD, SQLE
biosynthesis of saturated and n - 9 series of MUFA and PUFA	5	9	0.38976	1.5131E-5	5.7683E-4	ELOVL6, FADS1, FADS2, FASN, SCD
17-alpha-hydroxyprogesterone --- > 5alpha-androstanediol	3	5	0.21654	7.3989E-4	0.01569	AKR1C1 (ENSG00000187134), AKR1C2 (ENSG00000151632), SRD5A3
acetyl-CoA, malonyl-CoA --- > lignoceric acid	3	5	0.21654	7.3989E-4	0.01569	ELOVL6, FADS2, FASN
HMGCR regulation	9	65	2.81496	0.00158	0.02785	EGFR, FDFT1, FDPS, HMGCS1, IDI1, INSIG1, LSS, MVD, SQLE
Pathway fragments up-regulated in RS						
Aurora-B cell cycle regulation	17	55	4.09011	1.676E-7	4.0727E-5	BIRC5, BUB1, BUB1B, CCNB1, CCNB2, CDC20, CDCA8, CDK1, CENPE, CUL1, INCENP, MAD2L1, PLK1, TTK, UBB, UBE2C, ZC3HC1
Cdk1, Plk1 ---/ cyclin B	5	5	0.37183	2.1527E-6	1.7437E-4	CCNB1, CDC20, CDK1, CKS1B, PLK1
Plk1 --- > Bub1	5	5	0.37183	2.1527E-6	1.7437E-4	BUB1, CCNB1, CCNB2, CDK1, PLK1
Plk1 --- > INCENP	5	6	0.44619	1.2138E-5	7.3737E-4	CCNB1, CCNB2, CDK1, INCENP, PLK1
Plk1 activation and substrates	9	24	1.78478	2.7847E-5	0.00135	BRCA2, CCNB1, CCNB2, CDK1, KIF23, PLK1, PRKACB, RAD51, STK10
CENP-E --- > BubR1	5	7	0.52056	3.9925E-5	0.00162	BUB1, BUB1B, CENPE, MAD2L1, TTK
cyclosome regulation	16	75	5.57743	7.6369E-5	0.00265	CCNA2, CCNB1, CCNB2, CDC20, CDK1, CKS1B, CUL1, FBXO5, MAD2L1, NDC80, PLK1, SKP2, UBB, UBE2C, UBE2E2, UBE2S
cyclosome regulatory network	16	77	5.72616	1.0692E-4	0.00289	CCNA2, CCNB1, CCNB2, CDC20, CDK1, CKS1B, CUL1, FBXO5, MAD2L1, NDC80, PLK1, SKP2, UBB, UBE2C, UBE2E2, UBE2S
Cdc20 ubiquitination	8	22	1.63605	1.03E-4	0.00289	BUB1B, CCNB1, CDC20, CDK1, CKS1B, MAD2L1, UBB, UBE2C
Cdc20 deubiquitination	8	23	1.71041	1.4802E-4	0.0036	BUB1B, CCNB1, CDC20, CDK1, CKS1B, MAD2L1, UBB, UBE2C
Plk1 cell cycle regulation	12	52	3.86702	2.8909E-4	0.00585	BRCA2, CCNB1, CCNB2, CDK1, CUL1, FBXO5, KIF23, PLK1, PRKACB, RAD51, STK10, UBB
Metaphase to Anaphase transition	12	52	3.86702	2.8909E-4	0.00585	BUB1, BUB1B, CCNB1, CDC20, CDK1, CKS1B, FBXO5, MAD2L1, NEK2, PLK1, UBB, UBE2C
Bub1 --- > APC7	4	6	0.44619	3.9379E-4	0.00736	BUB1, BUB1B, CDC20, MAD2L1
S phase (Cdk2)	12	55	4.09011	5.0416E-4	0.00875	CCNA2, CDK1, CDKN3, CKS1B, CUL1, E2F3, E2F8, PPM1A, PPM1B, PPM1D, SKP2, UBB
ID complex deubiquitylation	4	7	0.52056	8.6562E-4	0.01402	CDK1, FANCD2, FANCI, UBB
borealin --- > Aurora-B	3	4	0.29746	0.00153	0.02323	BIRC5, CDCA8, INCENP
Pin1 --- > APP	3	5	0.37183	0.00361	0.05167	CCNB1, CCNB2, CDK1
Pathway fragments down-regulated in SIPS						
No significant findings						
Pathway fragments up-regulated in SIPS						
HMGCR regulation	21	65	6.1986	1.9691E-7	6.4979E-5	CAB39, CAB39L, CYP51A1, DHCR7, EGFR, FDFT1, FDPS, HMGCS1, IDI1, LIPA, PSMA7, PSMC1, PSMC4, PSMC5, PSMD11, PSMD2, PSMD8, SC5D, TM7SF2, UFD1L, VCP
acetyl-CoA, acetoacetyl-CoA --- > cholesterol, fatty acid	9	21	2.00262	5.8742E-5	0.00646	CYP51A1, DHCR7, FDFT1, FDPS, HMGCS1, IDI1, LIPA, SC5D, TM7SF2
cholesterol metabolism	9	21	2.00262	5.8742E-5	0.00646	CYP51A1, DHCR7, FDFT1, FDPS, HMGCS1, IDI1, LIPA, SC5D, TM7SF2
parkin associated pathways	15	65	6.1986	8.2044E-4	0.03437	CALM2, DNAJA1, HSPA8, PSMA7, PSMC1, PSMC4, PSMC5, PSMD11, PSMD2, PSMD8, TUBA1C, TUBB6, UBE2G1, UBE2L3, UBE2N
Mdm2 --- > p/CAF	8	23	2.19335	8.3317E-4	0.03437	PSMA7, PSMC1, PSMC4, PSMC5, PSMD11, PSMD2, PSMD8, TAF9 (ENSG00000085231)
HMGCR --- > 26S proteasome	9	28	2.67017	7.5931E-4	0.03437	PSMA7, PSMC1, PSMC4, PSMC5, PSMD11, PSMD2, PSMD8, UFD1L, VCP
ER-alpha ---CHIP--- > 26S proteasome	9	28	2.67017	7.5931E-4	0.03437	HSP90AA1, HSPA8, PSMA7, PSMC1, PSMC4, PSMC5, PSMD11, PSMD2, PSMD8
cofilin-1 degradation	8	22	2.09799	5.9124E-4	0.03437	CFL1, PSMA7, PSMC1, PSMC4, PSMC5, PSMD11, PSMD2, PSMD8
Smac ---/ cIAP-2	8	24	2.28871	0.00115	0.03446	BIRC3, PSMA7, PSMC1, PSMC4, PSMC5, PSMD11, PSMD2, PSMD8
E1 ---/ alpha-synuclein	8	24	2.28871	0.00115	0.03446	PSMA7, PSMC1, PSMC4, PSMC5, PSMD11, PSMD2, PSMD8, UBE2L3
NIK degradation	8	24	2.28871	0.00115	0.03446	PSMA7, PSMC1, PSMC4, PSMC5, PSMD11, PSMD2, PSMD8, TRAF3
Caspase network	17	82	7.81977	0.00137	0.03759	BID, BIRC3, CDC42, CFLAR, CRADD, DFFA, HSPD1, MCL1, PSMA7, PSMC1, PSMC4, PSMC5, PSMD11, PSMD2, PSMD8, UBE2L3, XIAP

Among the most downregulated signaling events significantly overrepresented in both bleomycin-induced and replicative type of senescence were GluR/AMPA receptor (GRIA1 isoforms), wnt/beta-catenin (TCF7L2/WNT2) and SDF-1 cascades (adjusted *p*-value range of <0.026 to < 0.05 for various fragments of these signaling pathways).

Upstream analysis aimed at identifying potential transcription factor binding sites (TFBSs) overrepresented in the promoters of differentially expressed genes commonly deregulated in both types of senescence was performed after filtration of gene expression levels by log fold change (FC) of 1.5 for up-regulated (*N* = 130 genes) and down-regulated (*N* = 177) genes, separately. The algorithm for transcription factor binding site (TFBS) enrichment analysis has been described in Kel et al. [14].

The outputs shown in Additional files 1 and 2 include the matrices of the hits which are over-represented in the Yes track (study set) versus the No track (background set), with only the overrepresented matrices with Yes-No ratio higher than 1 included, and the highest Yes-No ratios reflecting higher degrees of matches enrichment for the respective matrix in the Yes set. Matrix cut-off value were calculated and associated with the *P*-value score of enrichment as described before [14, 15].

Four homeobox genes, namely IRX2, HMX1, HDH, HOXC13 were binders for Top sites enriched in genes overexpressed in both bleomycin induced and replicative senescence phenotypes, while HOXB13, MAZ, GKLF, GLI, IK, SP1, PLZF, PBX were among transcription factors that preferentially bind to the sites located in genes downregulated both in RS and in SIPS.

### Genes uniquely involved in replicative senescence

A total of 1408 genes were upregulated and a total of 703 genes were downregulated in replicative senescence, but not in bleomycin induced senescence as compared to younger control fibroblasts. Functional analysis was performed for the lists of up- and downregulated genes separately, as described before.

The list of the signaling events significantly overrepresented in replicative senescence, but not in bleomycin induced senescence was represented entirely by various fragments of cyclosome regulatory network (adjusted *p*-values range of <4.1e-5 to < 0.023), with Top overrepresented being Aurora-B cell cycle regulation. The list of most significantly downregulated fragments centered around fatty acid anabolism, with an emphasis on biosynthesis of n-9 MUFAs and PUFAs, cholesterol metabolism and biosynthesis of estrogens (adjusted *p*-value range of <5.8e-4 to < 0.028).

Upstream analysis aimed at identifying potential TFBSs overrepresented in the promoters of differentially expressed genes uniquely deregulated in replicative senescence was performed after filtration of gene expression levels by log fold change (FC) of 1.5 for up-regulated (*N* = 1408 genes) and down-regulated (*N* = 703) genes, separately.

The outputs are shown in Additional files 3 and 4. Interestingly, lists of putative transcription factor candidates for being positive drivers for replicative senescence was very similar to that driving both types of senescence. In particular, homeobox genes IRX2, HMX1, HOXB13, HOXC13 (*p*-values range of E-39 to < E-25) were among Top positive regulators of replicative senescence. The only non-homeobox positive regulator identified at similar levels of confidence was promyelocytic leukemia zinc finger PLZF (e-31). Transcription factors HOXB13, IRX2, PLZF, HDX, DUXL, CDX2 and CPXH were among these that significantly preferred to bind promoters of genes downregulated in replicative senescence (*p*-values range of E-23 to < E-12).

### Genes uniquely involved in bleomycin-induced senescence

A total of 1358 genes were upregulated and a total of 1791 genes were downregulated in bleomycin induced, but not in replicative senescence as compared to younger control fibroblasts. Functional analysis was performed for the lists of up- and downregulated genes separately, as described before.

The signaling event significantly overrepresented in bleomycin induced, but not in replicative senescence was HMGCR regulation (adjusted *p*-value <6.5e^−5^), followed by two cholesterol biosynthesis network fragments (adjusted *p*-values <0.006 for each event evaluated separately), and a number of events with the participation of proteasome or ubiquitin ligases (adjusted *p*-values range of < 0.03 for each separate event).

Upstream analysis aimed at identifying potential TFBSs overrepresented in the promoters of differentially expressed genes uniquely up-regulated (*N* = 1358 genes) and down-regulated (*N* = 1791) genes in bleomycin-induced senescence was performed similarly to that for the genes deregulated in replicative senescence.

The outputs are shown in Additional files 5 and 6. List of putative transcription factor candidates for genes with increased expression in bleomycin-induced senescence included homeobox genes IRX2, CPHX and HDX as well as other types of transcriptional factors, namely Helios, RelA and HNF3B (*p*-values range of E-10 to < E-8). A list of transcription factor bindings sites in overrepresented genes downregulated in bleomycin induced senescence were MAZ (E-13) and GKLF (E-12).

### Master regulators orchestrating replicative and bleomycin-induced senescence

An analysis of DEGs upregulated in RS and in SIPS identified stromelysin and MGAT1 as master regulator molecules that influence the replicative senescence and bleomycin–induced senescence expression programs, respectively.

### Bridging senescence regulators to ospeopontin secretion

**Fig. 2:**
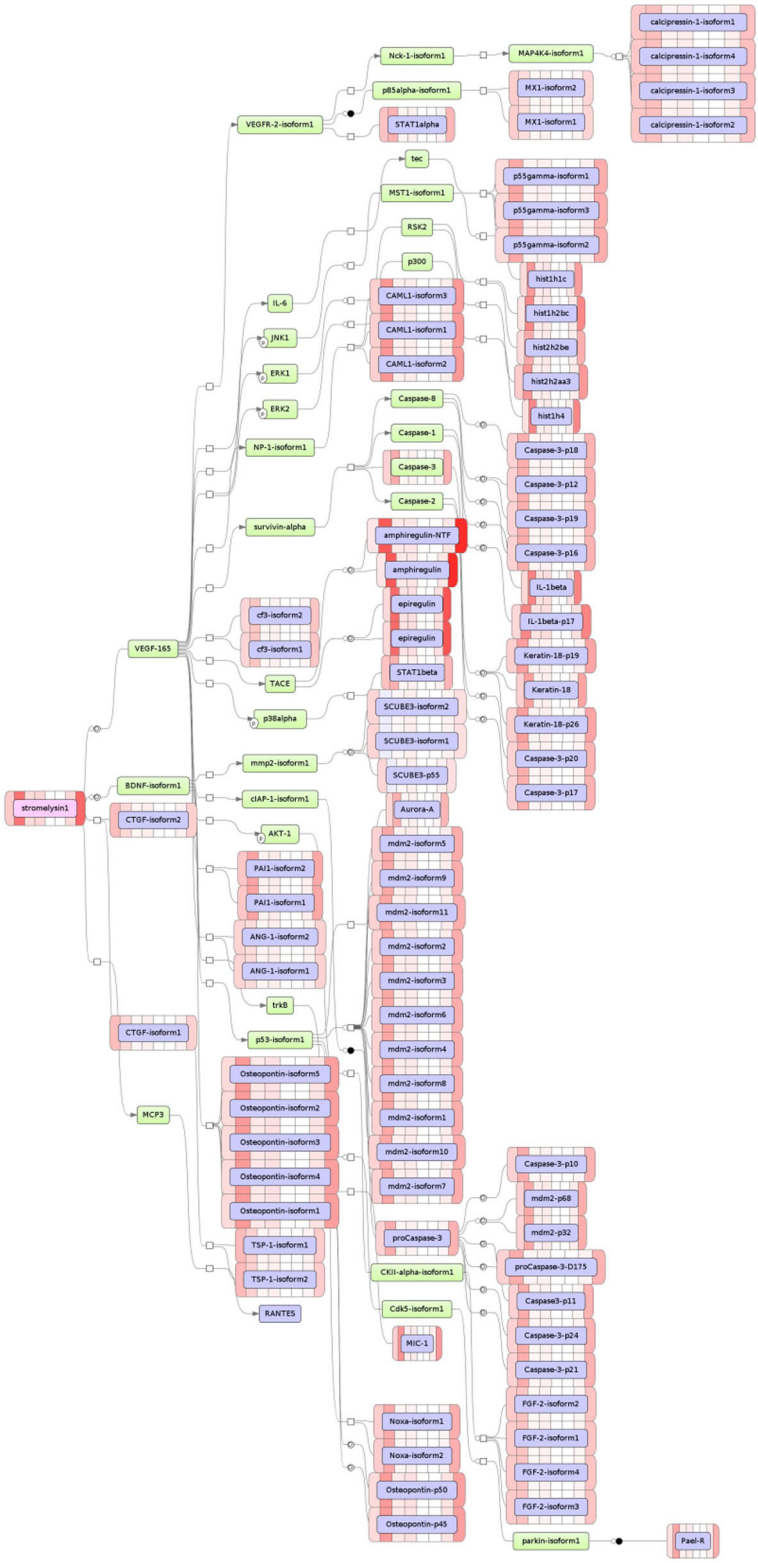
Hierarchically compiled output of an analysis for master regulators orchestrating gene expression program executed in replicative senescence. Stromelysin, the master regulator of this network, is highlighted in *red*, intermediate controllers that are added by GeneXPlain algorithm, a subset of input molecules is highlighted in *blue*. The intensity of the *pink*/*red* bars on a side of the molecule box represents the degree of overexpression for respective genes

In their previous publication, Pazolli et al. [8] identified *SPP1-*encoded osteopontin as a secreted driver for tumor cells growth that is provided by senescent fibroblast. To understand how senescence-wide targets highlighted by microarray analysis of senescent fibroblasts results in an increase in osteopontin secretion, a concise network was constructed using Shortest Path function in Pathway Studio software (Fig. 2). Iroquois Homeobox 2 (IRX2) and POU4F1 were highlighted as most plausible connecting signaling molecules.

## Discussion

Over past decade, transcriptome profiling efforts that employ either microarray or RNAseq have already generated enormous amounts of data, with respective data analysis often only scratching the surface [16, 17]. In many cases, high-quality datasets are generated to investigate specific hypothesis, and consequently, these datasets get analyzed in a particular way. At least in theory, the study design of these narrow-set, but technically sound experiments should allow extraction of additional information that could remain unrecovered at the moment that the main manuscript gets sent to the publishers [18].

In their 2009 paper, Pazolli et al. started to investigate the mechanisms of the manner in which senescent BJ fibroblasts stimulate the growth of preneoplastic cells in vitro and in vivo [8]. In their experiments, replicative senescent (RS) and stress-induced premature senescent (SIPS) fibroblasts were equally proficient at inducing the growth of HaCaT cells. Their study of fibroblasts/HaCaT xenografts in vivo arrived essentially at same results [8]. The authors subsequently hypothesized that growth-promoting activities of both types of senescent cells are maintained by a common core of genes. Based on that hypothesis, they embarked on microarray-driven dissection of secreted factors commonly produced by RS and SIPS fibroblasts. After a set of validation experiments in qRT-PCR and in-cell cultures, soluble protein osteopontin was highlighted as the protein of functional importance, and its gene, SPP1, was identified as a master regulator of a cancer niche environment [8]. An objective of the study achieved; however, the microarray dataset never got analyzed in larger context, i.e. for the purpose of direct comparison between RS and SIPS drivers.

In this study, we used the dataset of Pazolli et al., 2009 to extract the differentially expressed genes (DEGs) that differentiate the processes of RS and SIPS, to reconstruct relevant molecular cascades and to gain additional insights into popular cellular model of bleomycin induced senescence. Analysis of signaling events indicated that an involvement of caspase-3/keratin-18 pathway that is indicative of apoptotic rather than necrotic cell death [19] and an evolutionarily conserved serine/threonine kinase Aurora A/ MDM2 pathway essential for mitotic progression [20] was shared between both types of senescence. Observed upregulation of Aurora A is consistent with previously demonstrated increase in a number of aneuploid cells observed in ageing fibroblast cultures [21]. Our analysis also highlighted concerted alteration of glutamate, polyamine and choline metabolisms as well as wnt/β-catenin and SDF-1/CXCL12 cascades. All these findings are generally consistent with previous studies of various ageing fibroblasts both in culture and in human cohorts [22–24]. This consistency prompts us to stress on the high quality of the dataset of Pazolli et al., 2009 being analyzed.

An analysis aimed at identifying master regulator molecules that influence the replicative senescence and bleomycin–induced senescence expression programs, pointed at stromelysin/MMP3 and N-acetylglucosaminyltransferase enzyme MGAT1 that initiates the synthesis of hybrid and complex N-glycans as key orchestrating components in replicative senescence and in bleomycin-induced senescence, respectively (Fig. 2 and Additional file 7). Traditionally, MMP3 is seen as end-point biomarker or effector molecule associated with ageing in fibroblasts. However, in Hutchinson-Gilford progeria syndrome, there is a progressive loss of *MMP3* mRNA and protein expression [25]. Another study linked carrier status for *MMP3* 6A (rs3025058) allele to skin and lung aging [26]. Moreover, an exposure to MMP3 stimulates expression of Rac1b, a tumor-associated protein with cell-transforming properties that aids in bypassing replicative senescence [27] while driving motility and protumorigenic responses of the stroma [28]. Hence, there is an accumulation of evidence that stresses on an importance of MMP3 as a molecule of importance in replicative senescence that deserves additional investigations. An identification of MGAT1 that controls the synthesis the complex N-glycan sugars in the Golgi as the key regulator of SIPS is even more intriguing as there is strong associations between human plasma N-glycans and age [29, 30].

Specific question that we aimed to dissect was on the differences of the senescence programs executed in SIPS and RS. Indeed, our analysis showed that in RS fibroblasts, the list Top deregulated events is populated by fragments of Aurora-B driven cell cycle signaling that are accompanied by the suppression of anabolic branches of the fatty acids and estrogen metabolism. This may be interpreted as an execution of ordered senescence program that proceeds along with shutting down the metabolism on a way to the halt of mitotic progression and apoptosis that is being upregulated in both RS and SIPS. On the other end, in bleomycin exposed fibroblasts, Aurora-B signaling is deprioritized and the synthetic branches of cholesterol metabolism are upregulated, rather than downregulated, while proteasome/ ubiquitin ligase pathways of protein degradation are dominating the regulatory landscape. This picture is indicative that the cells are going down actively fighting overwhelming amounts of stress that is facilitating premature senescence of cells, but fail to completely activate orderly program of replicative senescence. Latter observation is consistent with activation of 26S proteasome and enhanced protein polyubiquitination previously observed in both idiopathic and bleomycin-induced pulmonary fibrosis [31]. Generalized mechanistic depiction of cellular processes common and differentiating RA and SIPS is presented at Fig. 2.

The list of the transcription factors capable of binding within the promoter regions of the genes that change their expression in either RS or SIPS was unusually enriched by the members of homeobox family, with particular emphasis on HMX1, IRX2, HDX and HOXC13. The possibility of an involvement of homeobox genes in ageing has been proposed earlier [32], with many homeobox containing TFs included in manually curated GenAge reference database [33]. Our findings indicate that the senescent program may be orchestrated by transcription factors (TFs) of Homeobox family at least in case of replicative senescence in vitro. On the other hand, promoters of genes that change their expression in bleomycin-induced senescence but not in replicatively senescent fibroblasts were enriched by binding sites for transcription factors Ikaros, RelA, HNF3B, GKLF and MAZ. Both RelA and GKLF are known stress-induced transcription regulators. RelA is the central player in the classical (or canonical) pathway of induction of NF-κB subunits that promotes senescence when activated in human lung fibroblasts exposed to ROS [34]. GKLF-deficient fibroblasts exposed to excessive levels of reactive oxygen species are more prone to become prematurely senescent than normal fibroblasts [35]. Moreover, yet another transcription factor, HNF3B/FOXA2 is epigenetically silenced in peroxide-stressed fibroblasts [36], therefore, an enrichment for binding sites for this factor in transcripts downregulated in bleomycin induced senescence is not surprising.

**Fig. 3:**
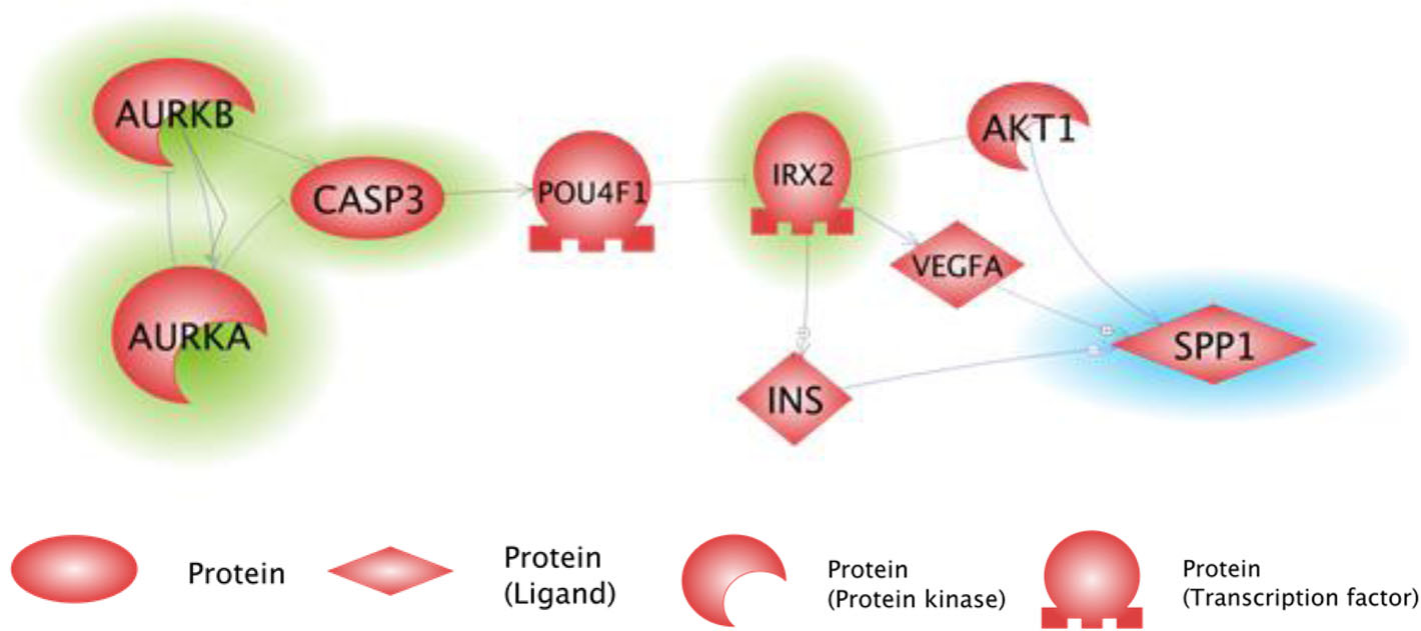
Pathway Studio guided network that describes regulatory connections between the deregulation of Aurora kinases, caspase-3 and osteopontin-encoding *SPP1*. Genes that were highlighted by either analysis of DEGs or by analysis of TFBS are highlighted in *green*. The gene of interests, *SPP1*, is highlighted in *blue*

SPP1-encoded osteopontin, a secreted stromal driver for tumor growth, is overexpressed by both RS and SIPS fibroblasts [8]. Concise network constructed using Shortest Path function in Pathway Studio software (Fig. 3) highlighted Iroquois Homeobox 2 (IRX2) and POU4F1 were highlighted as most likely signaling events to connect the DEGs identified by GeneXPlain-guided microarray analysis and osteopontin. In this network, suppression of Aurora kinases that normally monitor the mitotic checkpoint, centrosome separation and cytokinesis, cause catastrophic consequences and result in increase in apoptosis, thus, being in in agreement with recently published observations of senescent fibroblasts [37]. Apoptosis activated caspase-3 directly or indirectly eliminates POU4F1/Brn-3a, the prediction that is consistent with previous observation of enhanced apoptosis in the neurons derived from *Brn-3a* knockout mice [38]. Moreover, *POU4F1* gene is expressed in fibroblasts where it is required for proliferation, and cooperates with activated RAS/RAF signalling by reducing oncogene-induced senescence, consistent with its caspase-driven downregulation in both RS and SIPS [39]. In our network, POU4F1/Brn-3a suppresses transcription factor IRX2 that repeatedly showed up in lists of TF that recognize bindings sites differentially enriched in promoters of genes associated with fibroblast senescence. Caspase-3-driven removal of POU4F1 allows higher levels of IRX2 biosynthesis that is known for its ability to upregulate VEGF, metalloproteinases and other secreted molecules [40, 41].

An involvement of IRX2 in the transcription of osteopontin-encoding SPP1 gene was never evaluated in wet lab experiments; however, the knowledge-based algorithm identified IRX2 as positive regulator of *SPP1* expression by three independent molecular interaction events involving AKT1, VEGFA and INS. Moreover, marker co-expression pattern of *IRX2* and *SPP1* was observed during hair-cell development in the chick’s cochlea [42]. Two independent studies demonstrated that an expression of *IRX2* is commonly suppressed by DNA methylation of its promoter [43, 44], including its differential methylation noted in osteoarthritis and osteoporosis [45], two age-related diseases of the cartilage and the bone charactrized by changes in the levels of osteopontin secretion [46, 47]. As *IRX2* is strongly expression in human primary osteoblasts of the skeleton [48], its putative roles in SPP1 regulation in osteoarthritis and osteoporosis are worthy of investigation.

Importanttly, Pazolli and co-authors followed up on their own study that identified osteopontin as driver of tumor cell proliferation supplied by senescent stromal fibroblasts [8] and showed that the treatment with histone deacetylase (HDAC) inhibitors that reverse CpG methylation is sufficient to induce expression of osteopontin [49]. Moreover, an examination of PWM matches in the promoter of *SPP1* showed that it contains 25 sites for IRX2 binding within 1100 nucleotides located between positions −1000 to +100 relative to major transcription start site (TSS) for SPP1 gene (Additional file 8).

All this evidence adds up in favor of the hypothesis that SPP1/osteopontin expression may be controlled by IRX2, and that its derepression in senescent fibroblast aids in SIPS-dependent stromal activation that, in turn, stimulate the growth of tumor cells.

## Conclusions

Here we present a detailed comparison of stress/bleomycin induced and replicative senescence. We predicted the master regulatory molecules and transcription factors which play a key role in these two types of cell senescence, RS and SIPS. We showed that SIPS proceeds in cells that are actively fighting stress which facilitates premature senescence while failing to completely activate the orderly program of RS. Stromelysin/MMP3 and MGAT1 were identified as master regulators of RS and SIPS, respectively. We also demonstrated that promoters of genes differentially expressed in either RS or SIPS are unusually enriched by the binding sites for homeobox family proteins. Moreover, Iroquois Homeobox 2 (IRX2) was highlighted as a master regulator for the secretion of *SPP1*-encoded osteopontin, a stromal driver for tumor growth that is overexpressed by both RS and SIPS fibroblasts. The latter supports the hypothesis that senescence-specific de-repression of *SPP1* aids in SIPS-dependent stromal activation.

## Additional files

Additional file 1: Table S1. Transcription Factor Binding Sites within upstream regions of genes up-regulated in both types of senescence with log Fold Change > 2.0. (DOCX 23 kb)
Additional file 2: Table S2. Transcription Factor Binding Sites within upstream regions of genes down-regulated in both types of senescence with log Fold Change > 2.0. (DOCX 19 kb)
Additional file 3: Table S3. Transcription Factor Binding Sites within upstream regions of genes up-regulated in replicative senescence with log Fold Change > 1.5. (DOCX 27 kb)
Additional file 4: Table S4. Transcription Factor Binding Sites within upstream regions of genes down-regulated in replicative senescence with log Fold Change > 1.5. (DOCX 22 kb)
Additional file 5: Table S5. Transcription Factor Binding Sites within upstream regions of genes up-regulated in bleomycin induced senescence with log Fold Change > 1.5. (DOCX 24 kb)
Additional file 6: Table S6. Transcription Factor Binding Sites of Down-regulated genes with log Fold Change < − 1.5 threshold for bleomycin induced cell senescence. (DOCX 21 kb)
Additional file 7: Figure S1. Hierarchically compiled output of an analysis for master regulators orchestrating gene expression program executed in SIPS. MGAT1, the master regulator of this network, is highlighted in red, intermediate controllers that are added by GeneXPlain algorithm, a subset of input molecules is highlighted in blue. The intensity of the pink/red bars on a side of the molecule box represents the degree of overexpression for respective genes. (PNG 669 kb)
Additional file 8: Figure S2. IRX2 binding sites in the promoter of SPP1. (DOCX 180 kb)
