# Supplementary material for: Pathways of aging: comparative analysis of gene signatures in replicative senescence and stress induced premature senescence
Source: BMC Genomics. 2016 Dec 28;17(Suppl 14):1030. doi: 10.1186/s12864-016-3352-4 (PMC5249001; doi:10.1186/s12864-016-3352-4)
Supplement: Additional file 1: Table S1. — Transcription Factor Binding Sites within upstream regions of genes up-regulated in both types of senescence with log Fold Change > 2.0. (DOCX 23 kb) [file 12864_2016_3352_MOESM1_ESM.docx]

Supplementary Table S1: Transcription Factor Binding Sites within upstream regions of genes upregulated in both types of senescence with log Fold Change > 2.0.

| **ID** | **Yes density per 1000bp** | **No density per 1000bp** | **Yes-No ratio** | **Model cutoff** | **P-value** |
| --- | --- | --- | --- | --- | --- |
| V$NKX25_Q6 | 0.03286 | 0.00306 | 10.73494 | 0.9931 | 0.0103 |
| V$TBX5_Q2 | 0.03834 | 0.00612 | 6.26205 | 0.944 | 0.01296 |
| V$RORALPHA_Q4 | 0.08215 | 0.0153 | 5.36747 | 0.9661 | 4.2012E-4 |
| V$MRF2_01 | 0.04929 | 0.01224 | 4.0256 | 0.9253 | 0.01494 |
| V$SOX2_Q3_01 | 0.19168 | 0.06428 | 2.98193 | 0.9799 | 4.4995E-5 |
| V$CIZ_01 | 0.08215 | 0.03061 | 2.68373 | 0.999 | 0.0119 |
| V$REVERBALPHA_Q6 | 0.06572 | 0.02449 | 2.68373 | 0.923 | 0.02388 |
| V$TEF1_Q6_04 | 0.15882 | 0.06122 | 2.59428 | 0.9795 | 7.3828E-4 |
| V$ERALPHA_01 | 0.08762 | 0.03673 | 2.38554 | 0.8032 | 0.01741 |
| V$SRF_Q5_02 | 0.06572 | 0.02755 | 2.38554 | 0.9202 | 0.03782 |
| V$HNF4A_Q3 | 0.10405 | 0.04591 | 2.26627 | 0.9369 | 0.01346 |
| V$HSF1_01 | 0.26287 | 0.1255 | 2.09462 | 0.9711 | 3.7336E-4 |
| V$ZSCAN4_04 | 0.31216 | 0.15917 | 1.96119 | 0.9276 | 3.2726E-4 |
| V$LEF1_Q5_01 | 0.38335 | 0.20202 | 1.89759 | 0.9966 | 1.3972E-4 |
| V$TATA_01 | 0.85433 | 0.46832 | 1.82424 | 0.9413 | 1.0162E-7 |
| V$STAT1_Q6 | 0.08215 | 0.04591 | 1.78916 | 0.9915 | 0.07897 |
| V$ZFP105_04 | 0.33954 | 0.18978 | 1.78916 | 0.8666 | 8.6587E-4 |
| V$HNF6_Q4 | 0.43264 | 0.24181 | 1.78916 | 0.9235 | 1.8783E-4 |
| V$IRF1_Q5 | 0.12596 | 0.0704 | 1.78916 | 0.9829 | 0.03426 |
| V$POU6F1_02 | 0.50383 | 0.28466 | 1.76992 | 0.8469 | 7.6476E-5 |
| V$PBX_Q3 | 0.38335 | 0.21732 | 1.76396 | 0.895 | 5.4427E-4 |
| V$E2A_Q6_01 | 0.19168 | 0.11019 | 1.73946 | 0.9844 | 0.01377 |
| V$ETS_Q6 | 0.24096 | 0.14386 | 1.67496 | 0.999 | 0.00962 |
| V$HIC1_08 | 0.15882 | 0.09489 | 1.67373 | 0.9737 | 0.03166 |
| V$DLX3_02 | 0.15334 | 0.09183 | 1.66988 | 0.9955 | 0.03493 |
| V$REST_Q5 | 0.16429 | 0.10101 | 1.62651 | 0.9123 | 0.03639 |
| V$SF1_Q5_01 | 0.32859 | 0.20202 | 1.62651 | 0.96 | 0.00439 |
| V$POU2F1_Q6 | 0.78861 | 0.49281 | 1.60024 | 0.9335 | 3.0419E-5 |
| V$CDX2_Q5_02 | 0.99671 | 0.62749 | 1.58842 | 1 | 4.1793E-6 |
| V$HNF1A_Q4 | 0.57503 | 0.36425 | 1.57867 | 0.8927 | 4.5448E-4 |
| V$PIT1_Q6_01 | 1.23768 | 0.8142 | 1.52011 | 0.9359 | 2.7507E-6 |
| V$RFX1_01 | 0.35049 | 0.23263 | 1.50666 | 0.9156 | 0.01031 |
| V$DMRT4_01 | 0.16429 | 0.11019 | 1.49096 | 0.886 | 0.06862 |
| V$AP1_Q6_02 | 1.5115 | 1.05601 | 1.43133 | 0.9099 | 6.4332E-6 |
| V$AIRE_01 | 0.50383 | 0.37343 | 1.3492 | 0.9046 | 0.01847 |
| V$MEIS1_01 | 0.33406 | 0.24793 | 1.34739 | 0.9901 | 0.04794 |
| V$CEBPA_Q6 | 1.47864 | 1.10193 | 1.34187 | 0.9733 | 1.6935E-4 |
| V$RELA_Q6 | 0.32859 | 0.24487 | 1.34187 | 0.9255 | 0.05176 |
| V$GEN_INI_B | 0.49836 | 0.37343 | 1.33453 | 0.9914 | 0.02267 |
| V$BLIMP1_Q4 | 0.54217 | 0.41016 | 1.32184 | 0.9531 | 0.02136 |
| V$CPHX_01 | 3.83899 | 2.92317 | 1.3133 | 0.7443 | 3.1361E-8 |
| V$GATA_Q6 | 1.22125 | 0.93358 | 1.30814 | 0.9789 | 0.00143 |
| V$FPM315_01 | 0.47645 | 0.36425 | 1.30804 | 0.9362 | 0.03417 |
| V$SIX1_01 | 3.35159 | 2.59259 | 1.29276 | 0.7544 | 8.9365E-7 |
| V$TBX5_01 | 0.77218 | 0.59994 | 1.2871 | 0.9 | 0.01334 |
| V$ISL1_Q3 | 0.63527 | 0.49587 | 1.28112 | 0.9864 | 0.02474 |
| V$MZF1_Q5 | 1.16648 | 0.91521 | 1.27455 | 0.9856 | 0.00411 |
| V$ZNF333_01 | 2.11939 | 1.67432 | 1.26582 | 1 | 2.4103E-4 |
| V$DRI1_01 | 0.79956 | 0.63361 | 1.26192 | 1 | 0.01848 |
| V$CRX_Q4_01 | 0.66265 | 0.52648 | 1.25865 | 1 | 0.03079 |
| V$NF1_Q6 | 1.63198 | 1.31007 | 1.24572 | 0.9566 | 0.00215 |
| V$HNF3B_Q6 | 0.79409 | 0.63973 | 1.24128 | 0.9678 | 0.02647 |
| V$CDPCR1_01 | 4.89047 | 3.94245 | 1.24046 | 0.7788 | 4.9915E-7 |
| V$LRH1_Q5_01 | 0.56407 | 0.45914 | 1.22855 | 0.9727 | 0.062 |
| V$RBPJK_01 | 0.72289 | 0.59076 | 1.22367 | 0.8594 | 0.04248 |
| V$P53_Q3 | 1.35268 | 1.10805 | 1.22078 | 0.9531 | 0.00908 |
| V$HOXC13_01 | 8.06134 | 6.61769 | 1.21815 | 0.7331 | 3.6592E-9 |
| V$CDX2_01 | 2.07558 | 1.70493 | 1.2174 | 0.831 | 0.00185 |
| V$SOX10_Q3 | 0.96386 | 0.79278 | 1.2158 | 0.9811 | 0.0261 |
| V$CP2_Q6 | 1.02957 | 0.85399 | 1.2056 | 0.982 | 0.02712 |
| V$BBX_03 | 0.96933 | 0.80808 | 1.19955 | 0.8292 | 0.03469 |
| V$IPF1_Q5 | 2.79299 | 2.34466 | 1.19121 | 0.9544 | 0.00128 |
| V$AML3_Q6 | 0.7667 | 0.64585 | 1.18712 | 0.9256 | 0.06506 |
| V$RUSH1A_02 | 3.08872 | 2.61096 | 1.18298 | 0.9765 | 0.00114 |
| V$MAF_Q4 | 1.27054 | 1.07744 | 1.17922 | 0.8998 | 0.02881 |
| V$HELIOSA_02 | 9.71522 | 8.24916 | 1.17772 | 0.7988 | 5.673E-8 |
| V$DUXL_01 | 10.5586 | 8.99602 | 1.1737 | 0.6877 | 3.0056E-8 |
| V$MAFA_Q4 | 0.84337 | 0.71931 | 1.17247 | 0.9616 | 0.06971 |
| V$XVENT1_01 | 2.15225 | 1.83961 | 1.16995 | 0.8503 | 0.00876 |
| V$IRX2_01 | 21.30887 | 18.3165 | 1.16337 | 0.6537 | 1.4819E-13 |
| V$DBP_Q6 | 13.09419 | 11.37129 | 1.15151 | 0.8576 | 4.6619E-8 |
| V$NFAT1_Q4 | 1.25411 | 1.10193 | 1.1381 | 1 | 0.06891 |
| V$HMX1_02 | 24.85761 | 21.92837 | 1.13358 | 0.6375 | 2.556E-11 |
| V$NF1A_Q6_01 | 3.59255 | 3.17417 | 1.13181 | 0.9842 | 0.0072 |
| V$HDX_01 | 26.24863 | 23.20784 | 1.13102 | 0.6804 | 1.6668E-11 |
| V$TTF1_Q5_01 | 2.14129 | 1.91919 | 1.11573 | 0.9771 | 0.04822 |
| V$PAX_Q6 | 7.40964 | 6.65442 | 1.11349 | 0.7049 | 0.00105 |
| V$PLZF_02 | 8.73494 | 7.85124 | 1.11256 | 0.6736 | 4.5699E-4 |
| V$HOXD12_01 | 12.15225 | 11.00092 | 1.10466 | 0.6761 | 1.2645E-4 |
| V$HOXB13_01 | 12.05367 | 10.94276 | 1.10152 | 0.7188 | 1.9844E-4 |
| V$HMGIY_Q3 | 4.91785 | 4.47505 | 1.09895 | 0.8627 | 0.014 |
| V$FREAC3_01 | 3.41731 | 3.14662 | 1.08603 | 0.7484 | 0.05509 |
| V$NANOG_01 | 10.96386 | 10.16223 | 1.07888 | 0.7318 | 0.00389 |
| V$RHOX11_01 | 24.60022 | 23.1711 | 1.06168 | 0.683 | 7.8649E-4 |
