# Supplementary material for: Pathways of aging: comparative analysis of gene signatures in replicative senescence and stress induced premature senescence
Source: BMC Genomics. 2016 Dec 28;17(Suppl 14):1030. doi: 10.1186/s12864-016-3352-4 (PMC5249001; doi:10.1186/s12864-016-3352-4)
Supplement: Additional file 2: Table S2. — Transcription Factor Binding Sites within upstream regions of genes down-regulated in both types of senescence with log Fold Change > 2.0. (DOCX 19 kb) [file 12864_2016_3352_MOESM2_ESM.docx]

Supplementary Table S2:

Transcription Factor Binding Sites of Down-regulated genes with log Fold Change > 2.0 threshold for both types of Cell Senescence.

| **ID** | **Yes density per 1000bp** | **No density per 1000bp** | **Yes-No ratio** | **Model cutoff** | **P-value** |
| --- | --- | --- | --- | --- | --- |
| V$PBX_Q3 | 0.36932 | 0.01684 | 21.9375 | 0.9056 | 2.4016E-5 |
| V$DELTAEF1_01 | 0.14205 | 0.01684 | 8.4375 | 0.9921 | 0.02953 |
| V$RHOX11_01 | 0.25568 | 0.05051 | 5.0625 | 0.9085 | 0.00892 |
| V$TEF1_Q6_04 | 0.22727 | 0.05051 | 4.5 | 0.9801 | 0.01835 |
| V$CPHX_01 | 0.51136 | 0.11785 | 4.33929 | 0.8487 | 4.3688E-4 |
| V$DEAF1_02 | 0.14205 | 0.03367 | 4.21875 | 0.8268 | 0.07171 |
| V$IPF1_Q5 | 0.14205 | 0.03367 | 4.21875 | 0.9993 | 0.07171 |
| V$SIX1_01 | 0.14205 | 0.03367 | 4.21875 | 0.8741 | 0.07171 |
| V$CDX2_01 | 0.17045 | 0.05051 | 3.375 | 0.922 | 0.07142 |
| V$BLIMP1_Q4 | 0.28409 | 0.08418 | 3.375 | 0.9698 | 0.01984 |
| V$REST_Q5 | 0.3125 | 0.10101 | 3.09375 | 0.8913 | 0.01977 |
| V$FPM315_01 | 1.02273 | 0.43771 | 2.33654 | 0.9421 | 6.7991E-4 |
| V$AIRE_01 | 0.3125 | 0.13468 | 2.32031 | 0.9064 | 0.05395 |
| V$POU2F1_Q6 | 0.34091 | 0.15152 | 2.25 | 0.9355 | 0.05019 |
| V$EGR1_Q6 | 0.25568 | 0.11785 | 2.16964 | 1 | 0.09564 |
| V$ZSCAN4_04 | 0.25568 | 0.11785 | 2.16964 | 0.9108 | 0.09564 |
| V$GFI1_Q6_01 | 0.3125 | 0.15152 | 2.0625 | 0.982 | 0.08049 |
| V$CEBPA_Q6 | 1.13636 | 0.6229 | 1.82432 | 0.9729 | 0.00588 |
| V$MAZR_01 | 0.39773 | 0.21886 | 1.81731 | 0.9453 | 0.08621 |
| V$STAT1_Q6 | 0.42614 | 0.23569 | 1.80804 | 0.9678 | 0.0788 |
| V$HSF1_01 | 0.48295 | 0.26936 | 1.79297 | 0.9646 | 0.06605 |
| V$HNF3B_Q6 | 0.625 | 0.35354 | 1.76786 | 0.9627 | 0.04309 |
| V$MZF1_Q5 | 2.64205 | 1.51515 | 1.74375 | 0.9774 | 1.1999E-4 |
| V$HIC1_08 | 0.59659 | 0.35354 | 1.6875 | 0.9496 | 0.06164 |
| V$PIT1_Q6_01 | 0.51136 | 0.30303 | 1.6875 | 0.9362 | 0.08008 |
| V$AP2ALPHA_03 | 0.85227 | 0.50505 | 1.6875 | 0.8904 | 0.02912 |
| V$PLZF_02 | 3.77841 | 2.28956 | 1.65028 | 0.673 | 2.8419E-5 |
| V$CP2_Q6 | 1.07955 | 0.65657 | 1.64423 | 0.9823 | 0.01964 |
| V$RUSH1A_02 | 0.99432 | 0.60606 | 1.64062 | 0.9925 | 0.02492 |
| V$MAZ_Q6_01 | 6.07955 | 3.75421 | 1.61939 | 0.8848 | 3.5191E-7 |
| V$HOXB13_01 | 9.26136 | 5.77441 | 1.60386 | 0.709 | 8.1364E-10 |
| V$RNF96_01 | 0.53977 | 0.3367 | 1.60312 | 0.9757 | 0.09452 |
| V$MUSCLEINI_B | 0.9375 | 0.60606 | 1.54688 | 0.8956 | 0.04604 |
| V$CDPCR1_01 | 2.72727 | 1.78451 | 1.5283 | 0.7979 | 0.00173 |
| V$SRY_Q6 | 0.88068 | 0.58923 | 1.49464 | 0.9626 | 0.06653 |
| V$TATA_01 | 1.5625 | 1.06061 | 1.47321 | 0.8673 | 0.02288 |
| V$CDX2_Q5_02 | 0.90909 | 0.6229 | 1.45946 | 0.999 | 0.07471 |
| V$FREAC3_01 | 1.81818 | 1.24579 | 1.45946 | 0.7499 | 0.01707 |
| V$RREB1_01 | 4.48864 | 3.19865 | 1.40329 | 0.7482 | 0.00106 |
| V$SP1_Q6_01 | 7.67045 | 5.55556 | 1.38068 | 0.9155 | 5.591E-5 |
| V$PAX_Q6 | 3.09659 | 2.28956 | 1.35248 | 0.75 | 0.01156 |
| V$NF1A_Q6_01 | 2.01705 | 1.49832 | 1.34621 | 0.9889 | 0.0375 |
| V$GLI_Q3 | 13.60795 | 10.16835 | 1.33827 | 0.8804 | 1.3058E-6 |
| V$IK_Q5_01 | 13.18182 | 10.18519 | 1.29421 | 0.9357 | 1.8821E-5 |
| V$DUXL_01 | 5.28409 | 4.15825 | 1.27075 | 0.7031 | 0.00806 |
| V$IRX2_01 | 10.65341 | 8.45118 | 1.26058 | 0.6563 | 4.2101E-4 |
| V$HOXC13_01 | 9.09091 | 7.23906 | 1.25581 | 0.6738 | 0.00122 |
| V$GKLF_Q4 | 24.43182 | 19.7138 | 1.23933 | 0.9471 | 1.1413E-6 |
| V$CPBP_Q6 | 11.5625 | 9.44444 | 1.22426 | 1 | 0.00111 |
| V$DBP_Q6 | 14.09091 | 11.53199 | 1.2219 | 0.8522 | 4.0257E-4 |
| V$HELIOSA_02 | 7.72727 | 6.44781 | 1.19843 | 0.7947 | 0.01277 |
| V$HMX1_02 | 11.93182 | 10.15152 | 1.17537 | 0.6366 | 0.00622 |
| V$HDX_01 | 8.92045 | 7.72727 | 1.15441 | 0.721 | 0.0276 |
| V$P53_04 | 11.10795 | 9.88215 | 1.12404 | 0.7739 | 0.03974 |
| V$ZIC1_05 | 23.18182 | 21.49832 | 1.07831 | 0.7075 | 0.04878 |
