# Supplementary material for: Pathways of aging: comparative analysis of gene signatures in replicative senescence and stress induced premature senescence
Source: BMC Genomics. 2016 Dec 28;17(Suppl 14):1030. doi: 10.1186/s12864-016-3352-4 (PMC5249001; doi:10.1186/s12864-016-3352-4)
Supplement: Additional file 3: Table S3. — Transcription Factor Binding Sites within upstream regions of genes up-regulated in replicative senescence with log Fold Change > 1.5. (DOCX 27 kb) [file 12864_2016_3352_MOESM3_ESM.docx]

**Supplementary Table S3:** Transcription Factor Binding Sites within upstream regions of genes upregulated in replicative senescence with log Fold Change > 1.5

| **ID** | **Yes density per 1000bp** | **No density per 1000bp** | **Yes-No ratio** | **Model cutoff** | **P-value** |
| --- | --- | --- | --- | --- | --- |
| V$OSR1_03 | 0.03565624 | 0.003060912 | 11.64705882 | 0.9927 | 0.008 |
| V$ZBRK1_01 | 0.023767083 | 0.003060912 | 7.764705882 | 0.9576 | 0.048 |
| V$MAZR_01 | 0.130718954 | 0.036730946 | 3.558823529 | 0.9628 | 2.49E-04 |
| V$CIZ_01 | 0.166369578 | 0.052035507 | 3.197231834 | 0.9988 | 1.01E-04 |
| V$RORALPHA_Q4 | 0.047534165 | 0.015304561 | 3.105882353 | 0.966 | 0.039 |
| V$PBX_Q3 | 0.077243018 | 0.027548209 | 2.803921569 | 0.9468 | 0.0139 |
| V$DMRT4_01 | 0.213903743 | 0.076522804 | 2.795294118 | 0.8964 | 5.56E-05 |
| V$REVERBALPHA_Q6 | 0.065359477 | 0.024487297 | 2.669117647 | 0.9244 | 0.0278 |
| V$NF1A_Q6_01 | 0.231729055 | 0.094888277 | 2.442125237 | 1 | 1.58E-04 |
| V$HIC1_08 | 0.18419489 | 0.094888277 | 1.941176471 | 0.9749 | 0.0067 |
| V$HNF1A_Q4 | 0.683303624 | 0.367309458 | 1.860294118 | 0.8921 | 1.67E-06 |
| V$MYB_05 | 0.106951872 | 0.058157331 | 1.839009288 | 0.9022 | 0.046 |
| V$DLX3_02 | 0.166369578 | 0.091827365 | 1.811764706 | 0.9955 | 0.0172 |
| V$POU6F1_02 | 0.469399881 | 0.260177533 | 1.804152249 | 0.8495 | 1.24E-04 |
| V$BCL6_Q3_01 | 0.27332145 | 0.15610652 | 1.750865052 | 0.9581 | 0.0043 |
| V$IRF1_Q5 | 0.130718954 | 0.076522804 | 1.708235294 | 0.9821 | 0.0469 |
| V$REST_01 | 0.118835413 | 0.070400979 | 1.68797954 | 0.8135 | 0.0604 |
| V$GATA_Q6 | 1.37849079 | 0.844811754 | 1.631713555 | 0.9841 | 3.50E-08 |
| V$POU2F1_Q6 | 1.628045157 | 1.007040098 | 1.616663687 | 0.8758 | 4.05E-09 |
| V$ARID5A_03 | 1.527035056 | 0.945821855 | 1.614505997 | 0.8788 | 1.29E-08 |
| V$HSF1_01 | 1.057635175 | 0.667278849 | 1.584997302 | 0.9392 | 4.13E-06 |
| V$HNF6_Q4 | 0.404040404 | 0.260177533 | 1.552941176 | 0.9191 | 0.0047 |
| V$PIT1_Q6_01 | 1.443850267 | 0.942760943 | 1.531512605 | 0.933 | 5.60E-07 |
| V$BBX_03 | 1.241830065 | 0.814202632 | 1.525210084 | 0.829 | 3.91E-06 |
| V$HSF1_02 | 0.213903743 | 0.140801959 | 1.519181586 | 0.8377 | 0.0396 |
| V$CDC5_01 | 0.326797386 | 0.217324763 | 1.503728252 | 0.8522 | 0.0153 |
| V$E2A_Q6_01 | 0.160427807 | 0.107131925 | 1.497478992 | 0.9845 | 0.07489 |
| V$ZNF333_01 | 2.465834819 | 1.674318947 | 1.472739004 | 1 | 2.50E-09 |
| V$CDX2_Q5_02 | 0.91503268 | 0.627486991 | 1.458249641 | 1 | 2.83E-04 |
| V$FPM315_01 | 0.5466429 | 0.382614019 | 1.428705882 | 0.9348 | 0.00613 |
| V$PLZF_02 | 11.28936423 | 7.903275176 | 1.428441245 | 0.6731 | 1.17E-31 |
| V$HMGIY_Q3 | 6.417112299 | 4.536271809 | 1.414622529 | 0.8622 | 5.99E-18 |
| V$LEF1_Q5_01 | 0.683303624 | 0.486685032 | 1.40399556 | 0.9954 | 0.0036 |
| V$ISL1_Q3 | 0.689245395 | 0.495867769 | 1.389978214 | 0.9864 | 0.0044 |
| V$IPF1_Q5 | 3.161021985 | 2.274257729 | 1.389913704 | 0.957 | 5.76E-09 |
| V$AP1_Q6_02 | 2.501485443 | 1.808999082 | 1.382800836 | 0.9025 | 2.91E-07 |
| V$XVENT1_01 | 3.743315508 | 2.708907254 | 1.381854437 | 0.8341 | 4.91E-10 |
| V$TATA_01 | 4.812834225 | 3.483318029 | 1.381680968 | 0.856 | 2.04E-12 |
| V$DRI1_01 | 0.873440285 | 0.633608815 | 1.378516624 | 1 | 0.0019 |
| V$HNF3B_Q6 | 1.782531194 | 1.297826752 | 1.373473918 | 0.9559 | 1.85E-05 |
| V$MAZ_Q6_01 | 0.944741533 | 0.688705234 | 1.371764706 | 0.9234 | 0.00148 |
| V$CDX2_01 | 2.263814617 | 1.662075298 | 1.362040949 | 0.8322 | 2.77E-06 |
| V$ZFP105_04 | 2.953060012 | 2.182430364 | 1.353106179 | 0.7897 | 1.76E-07 |
| V$CRX_Q4_01 | 0.707070707 | 0.52647689 | 1.343023256 | 1 | 0.00838 |
| V$SOX10_Q3 | 0.926916221 | 0.694827058 | 1.334024359 | 0.9826 | 0.00352 |
| V$BBX_04 | 2.483660131 | 1.867156413 | 1.330183221 | 0.8125 | 4.98E-06 |
| V$DBP_Q6 | 2.471776589 | 1.870217325 | 1.321652065 | 0.9477 | 8.04E-06 |
| V$STAT1_Q6 | 0.606060606 | 0.465258647 | 1.302631579 | 0.9604 | 0.02347 |
| V$CDPCR1_01 | 5.098039216 | 3.948576676 | 1.291108071 | 0.7785 | 5.23E-09 |
| V$FREAC3_01 | 3.648247178 | 2.837465565 | 1.285741481 | 0.7542 | 9.68E-07 |
| V$NFAT1_Q4 | 1.414141414 | 1.101928375 | 1.283333333 | 1 | 0.00175 |
| V$HMX1_02 | 28.49079026 | 22.29874503 | 1.277685817 | 0.6363 | 3.45E-39 |
| V$CEBPA_Q6 | 2.10932858 | 1.686562596 | 1.250667236 | 0.9707 | 6.27E-04 |
| V$HOXC13_01 | 23.53535354 | 18.83073156 | 1.249837451 | 0.6625 | 1.11E-27 |
| V$IRX2_01 | 22.56684492 | 18.17569636 | 1.241594516 | 0.654 | 3.04E-25 |
| V$HOXB13_01 | 32.2459893 | 25.97796143 | 1.241282515 | 0.666 | 3.58E-35 |
| V$DUXL_01 | 14.14735591 | 11.53045608 | 1.226955449 | 0.6728 | 5.19E-15 |
| V$SOX2_Q3_01 | 1.960784314 | 1.600857055 | 1.224834102 | 0.9282 | 0.0023 |
| V$RUSH1A_02 | 5.359477124 | 4.383226201 | 1.222724285 | 0.9683 | 1.49E-06 |
| V$CREBP1_01 | 5.294117647 | 4.361799816 | 1.21374613 | 0.7391 | 3.74E-06 |
| V$TEF1_Q6_04 | 1.319073084 | 1.089684726 | 1.210508923 | 0.9039 | 0.0147 |
| V$NKX25_Q6 | 1.170528818 | 0.973370064 | 1.202552719 | 0.9584 | 0.0240 |
| V$SRY_Q6 | 1.865715983 | 1.554943373 | 1.199861047 | 0.9626 | 0.0064 |
| V$HOXD12_01 | 22.95306001 | 19.1429446 | 1.199034971 | 0.6348 | 7.32E-19 |
| V$HDX_01 | 26.32204397 | 22.01714111 | 1.195525061 | 0.6847 | 9.25E-21 |
| V$HELIOSA_02 | 7.593582888 | 6.41567187 | 1.183599012 | 0.8143 | 1.31E-06 |
| V$CPHX_01 | 19.16221034 | 16.29323538 | 1.176083809 | 0.6363 | 2.87E-13 |
| V$MZF1_Q5 | 1.069518717 | 0.915212733 | 1.16860122 | 0.9856 | 0.055 |
| V$SIX1_01 | 17.92632204 | 15.69023569 | 1.142514517 | 0.6493 | 4.19E-09 |
| V$TTF1_Q5_01 | 2.192513369 | 1.919191919 | 1.142414861 | 0.9771 | 0.0234 |
| V$HOMEZ_01 | 21.79441474 | 19.35414754 | 1.126084975 | 0.6385 | 6.72E-09 |
| V$RHOX11_01 | 24.75341652 | 22.85583104 | 1.08302413 | 0.6839 | 2.07E-05 |

|  |  |  |  |  |  |
| --- | --- | --- | --- | --- | --- |
|  |  |  |  |  |  |
|  |  |  |  |  |  |
|  |  |  |  |  |  |
|  |  |  |  |  |  |
|  |  |  |  |  |  |
|  |  |  |  |  |  |
|  |  |  |  |  |  |
|  |  |  |  |  |  |
|  |  |  |  |  |  |
|  |  |  |  |  |  |
|  |  |  |  |  |  |
|  |  |  |  |  |  |
|  |  |  |  |  |  |
|  |  |  |  |  |  |
|  |  |  |  |  |  |
|  |  |  |  |  |  |
|  |  |  |  |  |  |
|  |  |  |  |  |  |
|  |  |  |  |  |  |
|  |  |  |  |  |  |
|  |  |  |  |  |  |
|  |  |  |  |  |  |
|  |  |  |  |  |  |
|  |  |  |  |  |  |
|  |  |  |  |  |  |
|  |  |  |  |  |  |
|  |  |  |  |  |  |
|  |  |  |  |  |  |
|  |  |  |  |  |  |
|  |  |  |  |  |  |
|  |  |  |  |  |  |
|  |  |  |  |  |  |
|  |  |  |  |  |  |
|  |  |  |  |  |  |
|  |  |  |  |  |  |
|  |  |  |  |  |  |
|  |  |  |  |  |  |
|  |  |  |  |  |  |
|  |  |  |  |  |  |
|  |  |  |  |  |  |
|  |  |  |  |  |  |
|  |  |  |  |  |  |
|  |  |  |  |  |  |
|  |  |  |  |  |  |
|  |  |  |  |  |  |
|  |  |  |  |  |  |
|  |  |  |  |  |  |
|  |  |  |  |  |  |
|  |  |  |  |  |  |
|  |  |  |  |  |  |
|  |  |  |  |  |  |
|  |  |  |  |  |  |
|  |  |  |  |  |  |
|  |  |  |  |  |  |
|  |  |  |  |  |  |
|  |  |  |  |  |  |
|  |  |  |  |  |  |
|  |  |  |  |  |  |
|  |  |  |  |  |  |
|  |  |  |  |  |  |
|  |  |  |  |  |  |
|  |  |  |  |  |  |
|  |  |  |  |  |  |
|  |  |  |  |  |  |
|  |  |  |  |  |  |
|  |  |  |  |  |  |
|  |  |  |  |  |  |
|  |  |  |  |  |  |
|  |  |  |  |  |  |
|  |  |  |  |  |  |
|  |  |  |  |  |  |
|  |  |  |  |  |  |
