# Supplementary material for: Pathways of aging: comparative analysis of gene signatures in replicative senescence and stress induced premature senescence
Source: BMC Genomics. 2016 Dec 28;17(Suppl 14):1030. doi: 10.1186/s12864-016-3352-4 (PMC5249001; doi:10.1186/s12864-016-3352-4)
Supplement: Additional file 4: Table S4. — Transcription Factor Binding Sites within upstream regions of genes down-regulated in replicative senescence with log Fold Change > 1.5. (DOCX 22 kb) [file 12864_2016_3352_MOESM4_ESM.docx]

Supplementary table S4: Transcription Factor Binding Sites of Down-regulated genes with log Fold Change<- 1.5 threshold for replicative Cell Senescence

| **ID** | **Yes density per 1000bp** | **No density per 1000bp** | **Yes-No ratio** | **Model cutoff** | **P-value** |
| --- | --- | --- | --- | --- | --- |
| V$DLX3_02 | 0.32362 | 0.0551 | 5.87371 | 0.9979 | 1.74E-08 |
| V$OSR1_03 | 0.05178 | 0.00918 | 5.63876 | 0.9914 | 0.02856 |
| V$FPM315_01 | 0.09061 | 0.01837 | 4.93392 | 0.9973 | 0.0054 |
| V$BRCA_01 | 0.10356 | 0.02449 | 4.22907 | 1 | 0.00527 |
| V$GCM2_01 | 0.07767 | 0.01837 | 4.22907 | 0.9563 | 0.01563 |
| V$TBX5_01 | 0.03883 | 0.00918 | 4.22907 | 0.9957 | 0.08841 |
| V$MYB_05 | 0.10356 | 0.03061 | 3.38326 | 0.9223 | 0.01246 |
| V$HNF1A_Q4 | 0.32362 | 0.10101 | 3.20384 | 0.9207 | 2.45E-05 |
| V$AIRE_01 | 0.06472 | 0.02143 | 3.02077 | 0.9402 | 0.06148 |
| V$CIZ_01 | 0.19417 | 0.07346 | 2.64317 | 0.9985 | 0.0039 |
| V$SOX10_Q3 | 0.10356 | 0.03979 | 2.60251 | 0.9992 | 0.03382 |
| V$SOX2_Q3_01 | 0.3754 | 0.14692 | 2.55506 | 0.9628 | 1.15E-04 |
| V$PAX_Q6 | 0.19417 | 0.07958 | 2.43985 | 0.881 | 0.00671 |
| V$ISL1_Q3 | 0.20712 | 0.08571 | 2.41661 | 0.9961 | 0.00557 |
| V$DMRT4_01 | 0.4919 | 0.24793 | 1.98401 | 0.8637 | 6.00E-04 |
| V$CDX2_01 | 3.15854 | 1.68962 | 1.86937 | 0.8314 | 4.88E-15 |
| V$STAT1_Q6 | 0.90614 | 0.48669 | 1.86186 | 0.9589 | 2.25E-05 |
| V$HBP1_03 | 0.32362 | 0.18059 | 1.79198 | 0.9168 | 0.01256 |
| V$HSF1_01 | 1.19092 | 0.66728 | 1.78475 | 0.9393 | 5.21E-06 |
| V$PIT1_Q6_01 | 1.60516 | 0.90909 | 1.76567 | 0.9336 | 2.23E-07 |
| V$NF1A_Q6_01 | 0.40129 | 0.22957 | 1.74802 | 0.9979 | 0.00782 |
| V$HSF1_02 | 0.20712 | 0.11938 | 1.735 | 0.8545 | 0.04895 |
| V$ARID5A_03 | 0.32362 | 0.18672 | 1.73323 | 0.9661 | 0.01696 |
| V$CRX_Q4_01 | 0.89319 | 0.52648 | 1.69655 | 1 | 2.32E-04 |
| V$POU6F1_02 | 0.80258 | 0.47444 | 1.69163 | 0.8321 | 4.92E-04 |
| V$DRI1_01 | 1.06147 | 0.63361 | 1.67528 | 1 | 9.09E-05 |
| V$ZFP105_04 | 1.77344 | 1.06214 | 1.66969 | 0.8172 | 6.57E-07 |
| V$HNF3B_Q6 | 2.20062 | 1.33456 | 1.64895 | 0.9557 | 6.52E-08 |
| V$CEBPA_Q6 | 2.3689 | 1.47536 | 1.60564 | 0.9718 | 9.29E-08 |
| V$ZNF333_01 | 2.66663 | 1.67432 | 1.59267 | 1 | 2.54E-08 |
| V$CPHX_01 | 9.21671 | 5.85246 | 1.57484 | 0.7141 | 1.27E-23 |
| V$BBX_03 | 1.20387 | 0.76829 | 1.56695 | 0.8309 | 2.19E-04 |
| V$HNF6_Q4 | 0.44012 | 0.29079 | 1.51356 | 0.903 | 0.02738 |
| V$CP2_Q6 | 0.45307 | 0.30609 | 1.48018 | 0.994 | 0.03212 |
| V$POU2F1_Q6 | 1.52749 | 1.06214 | 1.43813 | 0.8737 | 5.56E-04 |
| V$HNF4A_Q3 | 0.47896 | 0.33364 | 1.43556 | 0.8845 | 0.03889 |
| V$FREAC3_01 | 4.55658 | 3.22008 | 1.41505 | 0.7474 | 2.59E-08 |
| V$HMGIY_Q3 | 6.74425 | 4.79951 | 1.4052 | 0.86 | 3.77E-11 |
| V$BLIMP1_Q4 | 0.32362 | 0.23263 | 1.39114 | 0.9634 | 0.09742 |
| V$HOXD12_01 | 3.22326 | 2.31711 | 1.39107 | 0.7496 | 6.53E-06 |
| V$TEF1_Q6_04 | 1.39804 | 1.02234 | 1.36748 | 0.9059 | 0.00342 |
| V$HOXB13_01 | 12.32347 | 9.02357 | 1.3657 | 0.7314 | 2.38E-16 |
| V$ETS_Q6 | 0.73785 | 0.54178 | 1.3619 | 0.9871 | 0.028 |
| V$TATA_01 | 4.16823 | 3.07928 | 1.35364 | 0.8652 | 2.36E-06 |
| V$IPF1_Q5 | 2.71841 | 2.02326 | 1.34358 | 0.9576 | 1.60E-04 |
| V$DUXL_01 | 10.34291 | 7.75941 | 1.33295 | 0.6971 | 2.86E-12 |
| V$GATA_Q6 | 1.47571 | 1.10805 | 1.33181 | 0.9763 | 0.00529 |
| V$PLZF_02 | 10.47236 | 7.92164 | 1.32199 | 0.673 | 7.85E-12 |
| V$CDX2_Q5_02 | 1.42393 | 1.08968 | 1.30674 | 0.999 | 0.00941 |
| V$NANOG_01 | 5.24265 | 4.02816 | 1.3015 | 0.7734 | 3.33E-06 |
| V$GFI1_Q6_01 | 0.4919 | 0.37955 | 1.29601 | 0.9779 | 0.0985 |
| V$SIX1_01 | 7.4821 | 5.78512 | 1.29334 | 0.7131 | 6.94E-08 |
| V$XVENT1_01 | 2.42068 | 1.88246 | 1.28591 | 0.8498 | 0.00181 |
| V$HMX1_02 | 28.2197 | 22.19773 | 1.27129 | 0.6366 | 3.90E-22 |
| V$IRX2_01 | 23.65018 | 18.65932 | 1.26747 | 0.653 | 1.91E-18 |
| V$LEF1_Q5_01 | 2.3689 | 1.87634 | 1.26251 | 0.961 | 0.00375 |
| V$RELA_Q6 | 0.64724 | 0.51729 | 1.2512 | 0.9051 | 0.09714 |
| V$HDX_01 | 30.0967 | 24.28834 | 1.23914 | 0.677 | 2.72E-19 |
| V$PBX_Q3 | 1.37215 | 1.11417 | 1.23154 | 0.8503 | 0.03547 |
| V$GEN_INI_B | 2.20062 | 1.79982 | 1.22269 | 0.9704 | 0.01286 |
| V$AP1_Q6_02 | 2.31712 | 1.89777 | 1.22097 | 0.9023 | 0.01146 |
| V$NF1_Q6 | 1.56632 | 1.28864 | 1.21548 | 0.9571 | 0.03472 |
| V$DBP_Q6 | 2.20062 | 1.81206 | 1.21443 | 0.9479 | 0.0155 |
| V$HOXC13_01 | 16.81532 | 13.89042 | 1.21057 | 0.6871 | 1.33E-09 |
| V$HIC1_08 | 1.82522 | 1.50903 | 1.20953 | 0.9014 | 0.02757 |
| V$CDPCR1_01 | 4.6213 | 3.87818 | 1.19162 | 0.7807 | 0.00216 |
| V$NFAT1_Q4 | 1.30743 | 1.10193 | 1.18649 | 1 | 0.07372 |
| V$RUSH1A_02 | 4.66013 | 4.00061 | 1.16486 | 0.9694 | 0.00617 |
| V$CREBP1_01 | 5.30737 | 4.57606 | 1.15981 | 0.7291 | 0.00467 |
| V$MYB_Q4 | 3.24915 | 2.8038 | 1.15884 | 0.9675 | 0.02215 |
| V$IK_Q5_01 | 2.67958 | 2.32935 | 1.15035 | 0.9765 | 0.04171 |
| V$MAFA_Q4 | 2.34301 | 2.08448 | 1.12403 | 0.9595 | 0.08873 |
| V$HELIOSA_02 | 11.36555 | 10.18365 | 1.11606 | 0.7827 | 0.0022 |
| V$TTF1_Q5_01 | 3.54688 | 3.2262 | 1.0994 | 0.9674 | 0.08735 |
| V$RHOX11_01 | 25.29417 | 23.52005 | 1.07543 | 0.682 | 0.00226 |
| V$HOMEZ_01 | 13.44966 | 12.56198 | 1.07066 | 0.6633 | 0.02626 |
