# Supplementary material for: Pathways of aging: comparative analysis of gene signatures in replicative senescence and stress induced premature senescence
Source: BMC Genomics. 2016 Dec 28;17(Suppl 14):1030. doi: 10.1186/s12864-016-3352-4 (PMC5249001; doi:10.1186/s12864-016-3352-4)
Supplement: Additional file 5: Table S5. — Transcription Factor Binding Sites within upstream regions of genes up-regulated in bleomycin induced senescence with log Fold Change > 1.5. (DOCX 24 kb) [file 12864_2016_3352_MOESM5_ESM.docx]

Supplementary Table S5:

Transcription Factor Binding Sites within upstream regions of genes upregulated in bleomycin induced senescence with log Fold Change > 1.5

| **ID** | **Yes density per 1000bp** | **No density per 1000bp** | **Yes-No ratio** | **Model cutoff** | **P-value** |
| --- | --- | --- | --- | --- | --- |
| V$HMGA2_01 | 0.03418 | 0.00612 | 5.58271 | 0.9052 | 0.03286 |
| V$NKX25_Q6 | 0.03418 | 0.00612 | 5.58271 | 0.992 | 0.03286 |
| V$ZSCAN4_04 | 0.04785 | 0.00918 | 5.21053 | 0.9798 | 0.01269 |
| V$HMGIY_Q3 | 0.07519 | 0.01837 | 4.09398 | 0.9715 | 0.00422 |
| V$TBX5_Q2 | 0.03418 | 0.00918 | 3.7218 | 0.9374 | 0.06551 |
| V$REVERBALPHA_Q6 | 0.08886 | 0.02449 | 3.62876 | 0.9218 | 0.0033 |
| V$REST_Q5 | 0.08202 | 0.02449 | 3.34962 | 0.9278 | 0.00675 |
| V$HNF3B_Q6 | 0.38961 | 0.13468 | 2.89286 | 0.9866 | 9.97E-08 |
| V$RORALPHA_Q4 | 0.08202 | 0.03061 | 2.6797 | 0.9634 | 0.01804 |
| V$ZFP105_04 | 0.08886 | 0.03367 | 2.6391 | 0.9252 | 0.01505 |
| V$RELA_Q6 | 0.59467 | 0.22957 | 2.59038 | 0.9269 | 1.54E-09 |
| V$GCM2_01 | 0.06152 | 0.02449 | 2.51222 | 0.9497 | 0.04853 |
| V$MEF2_03 | 0.10936 | 0.04591 | 2.38195 | 0.8994 | 0.01302 |
| V$CIZ_01 | 0.17088 | 0.07346 | 2.32613 | 0.9985 | 0.00264 |
| V$SOX2_Q3_01 | 0.30075 | 0.14692 | 2.04699 | 0.963 | 5.11E-04 |
| V$HSF1_02 | 0.06835 | 0.03367 | 2.03008 | 0.8779 | 0.08121 |
| V$SRF_Q5_02 | 0.06152 | 0.03061 | 2.00977 | 0.9182 | 0.09908 |
| V$XVENT1_01 | 0.34176 | 0.17141 | 1.99382 | 0.9143 | 3.35E-04 |
| V$HSF1_01 | 0.32809 | 0.17447 | 1.88049 | 0.9682 | 0.00103 |
| V$IRF1_Q5 | 0.12987 | 0.0704 | 1.84472 | 0.9824 | 0.03585 |
| V$E2A_Q6_01 | 0.84074 | 0.47138 | 1.78357 | 0.9766 | 1.68E-06 |
| V$INSM1_01 | 0.12987 | 0.07346 | 1.76786 | 0.9181 | 0.04605 |
| V$HNF1A_Q4 | 0.22556 | 0.13162 | 1.71376 | 0.9179 | 0.01453 |
| V$COE1_Q6 | 0.35543 | 0.21732 | 1.6355 | 0.9547 | 0.00514 |
| V$POU6F1_02 | 0.55366 | 0.33976 | 1.62955 | 0.841 | 6.45E-04 |
| V$CDPCR1_01 | 1.0458 | 0.64891 | 1.61161 | 0.8441 | 6.09E-06 |
| V$FPM315_01 | 0.93643 | 0.59382 | 1.57697 | 0.9217 | 3.71E-05 |
| V$PBX_Q3 | 0.78606 | 0.49893 | 1.57549 | 0.8738 | 1.50E-04 |
| V$AIRE_01 | 0.21189 | 0.13468 | 1.57331 | 0.9164 | 0.03638 |
| V$TBX5_01 | 0.51265 | 0.32752 | 1.56524 | 0.9566 | 0.00214 |
| V$BCL6_Q3_01 | 0.21189 | 0.13774 | 1.53835 | 0.9593 | 0.04367 |
| V$TTF1_Q5_01 | 0.30759 | 0.20508 | 1.49983 | 0.9997 | 0.02372 |
| V$ERALPHA_Q6_01 | 0.15721 | 0.10713 | 1.46745 | 1 | 0.09928 |
| V$ZIC1_05 | 0.25974 | 0.17753 | 1.46305 | 0.9133 | 0.04449 |
| V$CP2_Q6 | 1.30554 | 0.90603 | 1.44094 | 0.9812 | 6.32E-05 |
| V$CDX2_01 | 1.0663 | 0.74992 | 1.42188 | 0.8628 | 4.22E-04 |
| V$PIT1_Q6_01 | 1.13465 | 0.7989 | 1.42027 | 0.9363 | 2.95E-04 |
| V$MAFA_Q4 | 0.67669 | 0.48056 | 1.40812 | 0.9978 | 0.00509 |
| V$AP1_Q6_02 | 2.62474 | 1.8641 | 1.40805 | 0.9024 | 1.35E-07 |
| V$POU2F1_Q6 | 0.6972 | 0.49893 | 1.39739 | 0.9333 | 0.00531 |
| V$HNF6_Q4 | 0.36227 | 0.26018 | 1.39239 | 0.9191 | 0.03712 |
| V$MZF1_Q5 | 1.05947 | 0.76217 | 1.39007 | 0.9887 | 8.85E-04 |
| V$BLIMP1_Q4 | 0.45796 | 0.33058 | 1.38534 | 0.9569 | 0.02298 |
| V$RBPJK_01 | 0.73821 | 0.53872 | 1.3703 | 0.8626 | 0.00646 |
| V$BBX_03 | 0.92276 | 0.68871 | 1.33985 | 0.8353 | 0.00463 |
| V$TEF1_Q6_04 | 1.65414 | 1.23661 | 1.33764 | 0.9 | 2.38E-04 |
| V$LEF1_Q5_01 | 1.43541 | 1.07744 | 1.33224 | 0.9807 | 6.71E-04 |
| V$IPF1_Q5 | 0.73821 | 0.56321 | 1.31072 | 0.9909 | 0.01581 |
| V$MAF_Q4 | 1.52427 | 1.17539 | 1.29682 | 0.8977 | 0.0013 |
| V$GATA_Q6 | 1.51059 | 1.16621 | 1.29531 | 0.9745 | 0.00142 |
| V$ETS_Q6 | 1.3944 | 1.10499 | 1.26191 | 0.9779 | 0.00486 |
| V$SOX10_Q3 | 1.38072 | 1.09581 | 1.26001 | 0.976 | 0.00528 |
| V$EBOX_Q6_01 | 3.33561 | 2.67218 | 1.24828 | 0.901 | 5.80E-05 |
| V$SRY_Q6 | 1.43541 | 1.1509 | 1.2472 | 1 | 0.00626 |
| V$MYOGENIN_Q6_01 | 1.18934 | 0.96113 | 1.23744 | 1 | 0.01435 |
| V$NF1A_Q6_01 | 2.1121 | 1.73248 | 1.21912 | 0.9889 | 0.00306 |
| V$MEIS1_01 | 0.62201 | 0.51423 | 1.20959 | 0.9853 | 0.08234 |
| V$CEBPA_Q6 | 5.85099 | 4.84848 | 1.20677 | 0.9506 | 6.28E-06 |
| V$DELTAEF1_01 | 1.12098 | 0.93052 | 1.20469 | 0.9788 | 0.03143 |
| V$STAT1_Q6 | 0.71087 | 0.59382 | 1.19712 | 0.9504 | 0.07892 |
| V$HIC1_08 | 1.77033 | 1.4876 | 1.19006 | 0.9023 | 0.01367 |
| V$DUXL_01 | 13.82092 | 11.61922 | 1.18949 | 0.6724 | 2.30E-10 |
| V$HELIOSA_02 | 11.79084 | 9.99082 | 1.18017 | 0.7844 | 1.88E-08 |
| V$LRH1_Q5_01 | 0.82023 | 0.69789 | 1.17531 | 0.9643 | 0.08538 |
| V$CDX2_Q5_02 | 1.27136 | 1.08968 | 1.16672 | 0.999 | 0.04947 |
| V$PAX_Q6 | 7.79904 | 6.71258 | 1.16185 | 0.7045 | 2.46E-05 |
| V$IRX2_01 | 15.49556 | 13.40986 | 1.15553 | 0.6668 | 1.59E-08 |
| V$DBP_Q6 | 12.48804 | 10.85399 | 1.15055 | 0.8649 | 7.21E-07 |
| V$IK_Q5_01 | 2.66576 | 2.32935 | 1.14442 | 0.9765 | 0.0169 |
| V$CPHX_01 | 17.1702 | 15.00765 | 1.1441 | 0.6393 | 2.75E-08 |
| V$RUSH1A_02 | 5.02392 | 4.39241 | 1.14377 | 0.9682 | 0.00175 |
| V$FAC1_01 | 2.89815 | 2.55586 | 1.13392 | 0.8781 | 0.0194 |
| V$HDX_01 | 26.07656 | 23.05479 | 1.13107 | 0.681 | 3.97E-10 |
| V$NF1_Q6 | 1.6473 | 1.45699 | 1.13062 | 0.9549 | 0.06589 |
| V$NFAT1_Q4 | 1.24402 | 1.10193 | 1.12895 | 1 | 0.09953 |
| V$SIX1_01 | 15.42037 | 13.69146 | 1.12628 | 0.6593 | 2.53E-06 |
| V$TATA_01 | 3.86876 | 3.46495 | 1.11654 | 0.8564 | 0.01764 |
| V$NANOG_01 | 10.78606 | 9.68779 | 1.11337 | 0.734 | 2.81E-04 |
| V$GEN_INI_B | 3.92344 | 3.56596 | 1.10025 | 0.9608 | 0.03284 |
| V$HOXB13_01 | 7.71702 | 7.0401 | 1.09615 | 0.7439 | 0.00626 |
| V$HMX1_02 | 17.42994 | 16.24426 | 1.07299 | 0.6626 | 0.00186 |
| V$RHOX11_01 | 24.8257 | 23.23845 | 1.0683 | 0.6828 | 5.71E-04 |
| V$HOXC13_01 | 18.94053 | 17.98286 | 1.05325 | 0.6665 | 0.01273 |
