# Supplementary material for: Pathways of aging: comparative analysis of gene signatures in replicative senescence and stress induced premature senescence
Source: BMC Genomics. 2016 Dec 28;17(Suppl 14):1030. doi: 10.1186/s12864-016-3352-4 (PMC5249001; doi:10.1186/s12864-016-3352-4)
Supplement: Additional file 6: Table S6. — Transcription Factor Binding Sites of Down-regulated genes with log Fold Change < − 1.5 threshold for bleomycin induced cell senescence. (DOCX 21 kb) [file 12864_2016_3352_MOESM6_ESM.docx]

Supplementary table S6: Transcription Factor Binding Sites of Down-regulated genes with log Fold Change <- 1.5 threshold for bleomycin induced cell senescence

| **ID** | **Yes density per 1000bp** | **No density per 1000bp** | **Yes-No ratio** | **Model cutoff** | **P-value** |
| --- | --- | --- | --- | --- | --- |
| V$REVERBALPHA_Q6 | 0.02256 | 0.00306 | 7.36917 | 0.9674 | 0.07585 |
| V$HNF3B_Q6 | 0.10526 | 0.02143 | 4.91278 | 0.9917 | 3.75E-04 |
| V$XVENT1_01 | 0.07519 | 0.01837 | 4.09398 | 0.9421 | 0.00536 |
| V$ERALPHA_01 | 0.12782 | 0.03673 | 3.47989 | 0.8075 | 8.12E-04 |
| V$STAT1_Q6 | 0.06015 | 0.02143 | 2.8073 | 0.9943 | 0.0408 |
| V$RORALPHA_Q4 | 0.08271 | 0.03061 | 2.70203 | 0.9633 | 0.02015 |
| V$DLX3_02 | 0.22556 | 0.09183 | 2.45639 | 0.9955 | 4.70E-04 |
| V$HBP1_03 | 0.17293 | 0.0704 | 2.45639 | 0.9517 | 0.00208 |
| V$SOX2_Q3_01 | 0.32331 | 0.13468 | 2.40056 | 0.9643 | 4.47E-05 |
| V$MAZR_01 | 0.33083 | 0.16223 | 2.03927 | 0.9431 | 4.34E-04 |
| V$REST_01 | 0.09774 | 0.04897 | 1.99582 | 0.8257 | 0.05006 |
| V$NF1A_Q6_01 | 0.18797 | 0.09489 | 1.98096 | 1 | 0.00893 |
| V$CIZ_01 | 0.12782 | 0.06734 | 1.89812 | 0.9986 | 0.03614 |
| V$SIX1_01 | 0.24812 | 0.13774 | 1.80135 | 0.8634 | 0.00797 |
| V$POU6F1_02 | 0.45113 | 0.2663 | 1.69406 | 0.8489 | 0.00135 |
| V$CTCF_01 | 0.11278 | 0.06734 | 1.67481 | 0.9002 | 0.08716 |
| V$HNF1A_Q4 | 0.33835 | 0.20202 | 1.67481 | 0.9089 | 0.00578 |
| V$LEF1_Q5_01 | 0.33835 | 0.20202 | 1.67481 | 0.9966 | 0.00578 |
| V$ZSCAN4_04 | 0.6015 | 0.36119 | 1.66535 | 0.8951 | 3.58E-04 |
| V$EKLF_Q5_01 | 0.18797 | 0.11325 | 1.65972 | 0.9901 | 0.03588 |
| V$MAZ_Q6_01 | 2.86466 | 1.76921 | 1.61918 | 0.8881 | 5.26E-13 |
| V$INSM1_01 | 0.14286 | 0.08877 | 1.60936 | 0.916 | 0.07384 |
| V$DMRT4_01 | 0.3985 | 0.25406 | 1.56854 | 0.8607 | 0.00754 |
| V$GKLF_Q4 | 3.13534 | 2.01714 | 1.55435 | 0.9989 | 2.98E-12 |
| V$CDX2_01 | 0.34586 | 0.22345 | 1.54786 | 0.9017 | 0.01422 |
| V$FPM315_01 | 0.83459 | 0.54484 | 1.53179 | 0.9245 | 3.33E-04 |
| V$SRF_Q5_02 | 0.18045 | 0.11938 | 1.51163 | 0.884 | 0.07398 |
| V$RFX1_01 | 0.66917 | 0.44383 | 1.50772 | 0.9014 | 0.00169 |
| V$PIT1_Q6_01 | 0.69173 | 0.45914 | 1.50659 | 0.9511 | 0.00146 |
| V$MZF1_Q5 | 1.99248 | 1.32844 | 1.49987 | 0.9774 | 2.03E-07 |
| V$RNF96_01 | 1.90226 | 1.28558 | 1.47968 | 0.8908 | 8.10E-07 |
| V$BBX_03 | 0.2406 | 0.16529 | 1.45564 | 0.8921 | 0.06018 |
| V$HNF4A_Q3 | 0.30075 | 0.20814 | 1.44494 | 0.9063 | 0.04223 |
| V$GFI1_Q6_01 | 0.44361 | 0.31221 | 1.42085 | 0.9797 | 0.02081 |
| V$E2A_Q6_01 | 0.63158 | 0.44689 | 1.41327 | 0.9768 | 0.00774 |
| V$CP2_Q6 | 0.44361 | 0.3214 | 1.38026 | 0.9939 | 0.03034 |
| V$COE1_Q6 | 0.32331 | 0.23569 | 1.37175 | 0.9543 | 0.0607 |
| V$LRH1_Q5_01 | 0.26316 | 0.19284 | 1.36466 | 0.9828 | 0.08728 |
| V$PBX_Q3 | 3.08271 | 2.30793 | 1.3357 | 0.8277 | 1.95E-06 |
| V$RBPJK_01 | 0.93985 | 0.70401 | 1.335 | 0.8498 | 0.00602 |
| V$SP1_Q6_01 | 3.56391 | 2.67218 | 1.33371 | 0.9072 | 3.83E-07 |
| V$CPHX_01 | 2.24812 | 1.71717 | 1.3092 | 0.758 | 1.19E-04 |
| V$HSF1_01 | 1.06767 | 0.82032 | 1.30152 | 0.9368 | 0.00702 |
| V$RHOX11_01 | 0.91729 | 0.70707 | 1.29731 | 0.8719 | 0.01245 |
| V$SOX10_Q3 | 0.78195 | 0.603 | 1.29677 | 0.9838 | 0.0197 |
| V$DBP_Q6 | 3.54887 | 2.75482 | 1.28824 | 0.9371 | 6.25E-06 |
| V$DRI1_01 | 0.81203 | 0.63361 | 1.2816 | 1 | 0.02227 |
| V$GCM2_01 | 0.54135 | 0.42241 | 1.2816 | 0.8949 | 0.05272 |
| V$GATA_Q6 | 1.37594 | 1.07438 | 1.28068 | 0.9784 | 0.0042 |
| V$CDPCR1_01 | 2.43609 | 1.91919 | 1.26933 | 0.8099 | 3.27E-04 |
| V$HIC1_08 | 1.90977 | 1.50903 | 1.26556 | 0.9014 | 0.00147 |
| V$ISL1_Q3 | 0.62406 | 0.49587 | 1.25852 | 0.9864 | 0.05242 |
| V$MYOGENIN_Q6_01 | 1.18797 | 0.96113 | 1.23602 | 1 | 0.01774 |
| V$RREB1_01 | 1 | 0.8142 | 1.2282 | 0.7974 | 0.03104 |
| V$IPF1_Q5 | 1.65414 | 1.36211 | 1.2144 | 0.9628 | 0.0109 |
| V$POU2F1_Q6 | 1.16541 | 0.96113 | 1.21255 | 0.8776 | 0.02885 |
| V$EGR1_Q6 | 1.33835 | 1.10499 | 1.21118 | 0.9123 | 0.02127 |
| V$IRX2_01 | 3.04511 | 2.52219 | 1.20733 | 0.776 | 0.00117 |
| V$TEF1_Q6_04 | 1.4812 | 1.23049 | 1.20375 | 0.9002 | 0.01919 |
| V$P53_Q3 | 1.36842 | 1.1509 | 1.189 | 0.9521 | 0.03161 |
| V$GLI_Q3 | 6.3609 | 5.39333 | 1.1794 | 0.8868 | 5.07E-05 |
| V$DUXL_01 | 6.27068 | 5.41169 | 1.15873 | 0.7159 | 2.73E-04 |
| V$CHCH_01 | 1.12782 | 0.97337 | 1.15867 | 1 | 0.07634 |
| V$AP1_Q6_02 | 1.21805 | 1.05601 | 1.15344 | 0.9099 | 0.07437 |
| V$BEN_01 | 8.84211 | 7.7288 | 1.14405 | 0.8701 | 8.35E-05 |
| V$CPBP_Q6 | 6.31579 | 5.53719 | 1.14061 | 1 | 9.45E-04 |
| V$NANOG_01 | 4.73684 | 4.15978 | 1.13872 | 0.7719 | 0.004 |
| V$IK_Q5_01 | 12.81203 | 11.25497 | 1.13834 | 0.9265 | 6.14E-06 |
| V$HDX_01 | 6.81955 | 6.1157 | 1.11509 | 0.7625 | 0.00363 |
| V$ZIC1_05 | 21.99248 | 20.73768 | 1.06051 | 0.6973 | 0.0042 |
| V$HMX1_02 | 17.37594 | 16.48913 | 1.05378 | 0.6617 | 0.01838 |
