# Supplementary figures and images for: Pathways of aging: comparative analysis of gene signatures in replicative senescence and stress induced premature senescence
Source: BMC Genomics. 2016 Dec 28;17(Suppl 14):1030. doi: 10.1186/s12864-016-3352-4 (PMC5249001; doi:10.1186/s12864-016-3352-4)

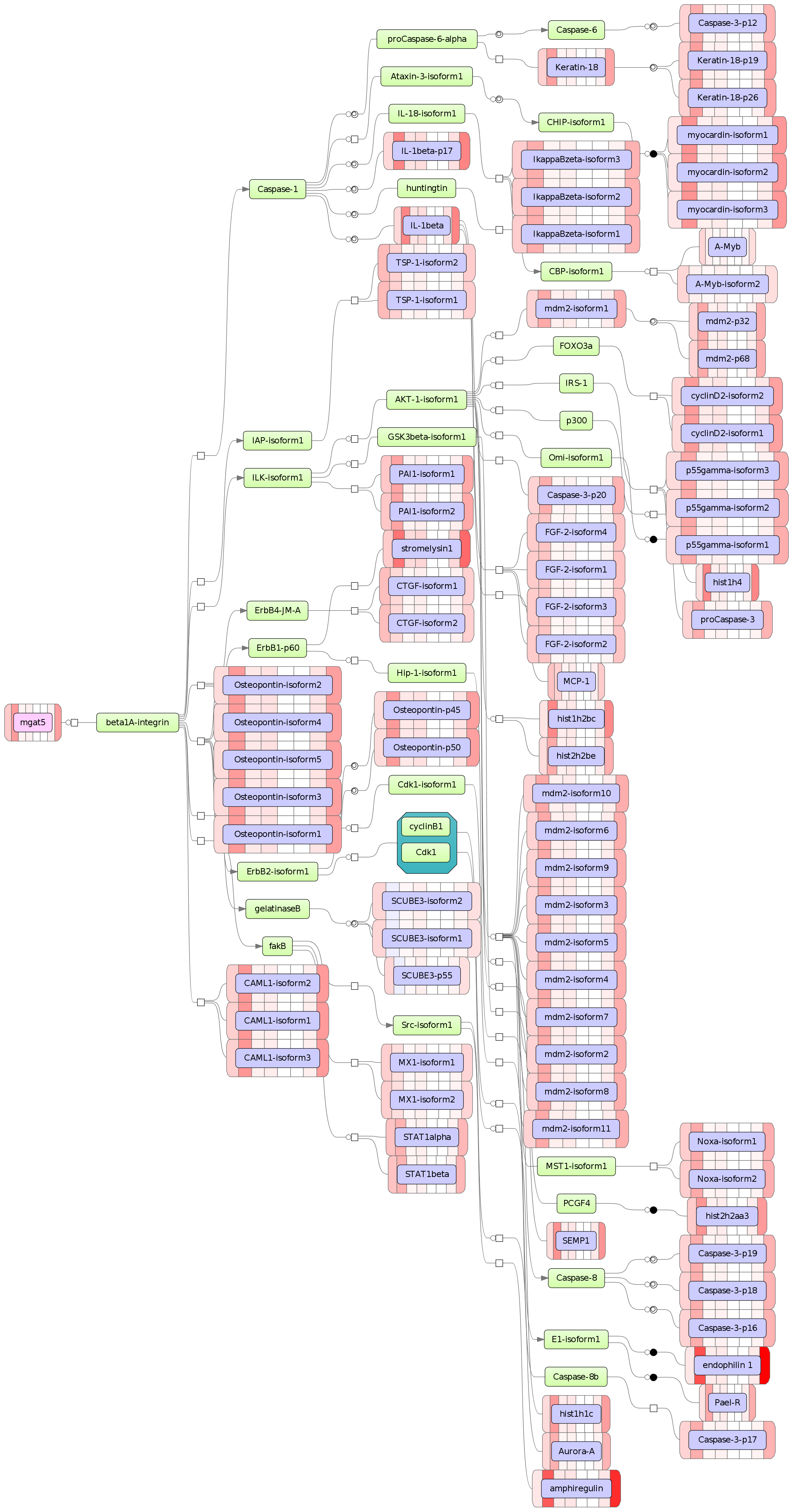

Supplement: Additional file 7: Figure S1. — Hierarchically compiled output of an analysis for master regulators orchestrating gene expression program executed in SIPS. MGAT1, the master regulator of this network, is highlighted in red, intermediate controllers that are added by GeneXPlain algorithm, a subset of input molecules is highlighted in blue. The intensity of the pink/red bars on a side of the molecule box represents the degree of overexpression for respective genes. (PNG 669 kb) [file 12864_2016_3352_MOESM7_ESM.png]
