# Supplementary material for: Pathways of aging: comparative analysis of gene signatures in replicative senescence and stress induced premature senescence
Source: BMC Genomics. 2016 Dec 28;17(Suppl 14):1030. doi: 10.1186/s12864-016-3352-4 (PMC5249001; doi:10.1186/s12864-016-3352-4)
Supplement: Additional file 8: Figure S2. — IRX2 binding sites in the promoter of SPP1. (DOCX 180 kb) [file 12864_2016_3352_MOESM8_ESM.docx]

***Gene: SPP1 ENSG00000118785 TSS 88896866***

***chr4:88,895,867-88,896,966***

*secreted phosphoprotein 1 [Source:HGNC Symbol;Acc:HGNC:11255]*

*Synonyms: BSPI, BNSP, OPN, ETA-1*

*IRX2 sites are shown as blue arrows.*

*
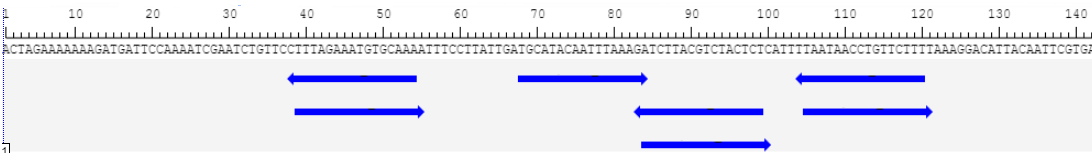
*

*
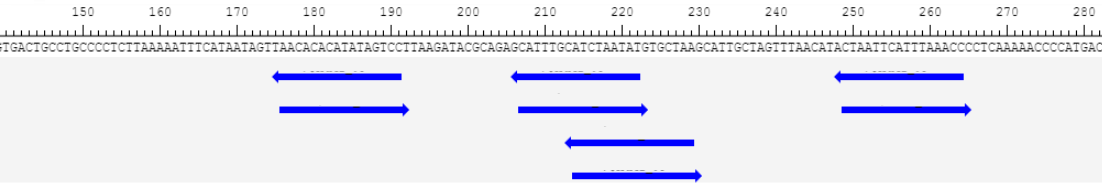
*

*
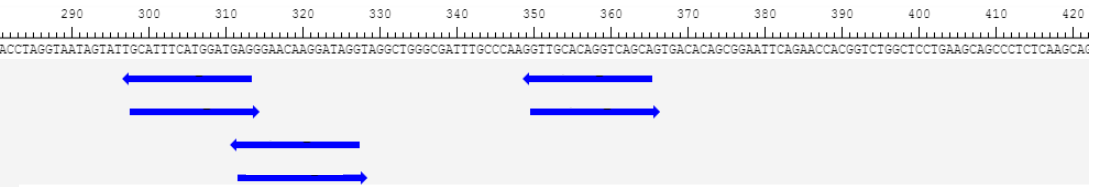
*

*
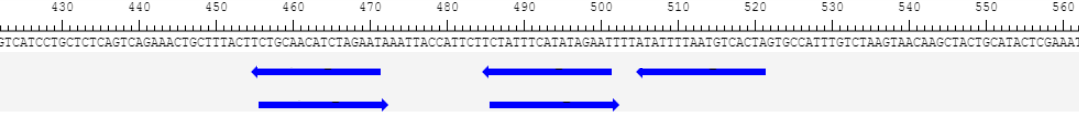
*

*
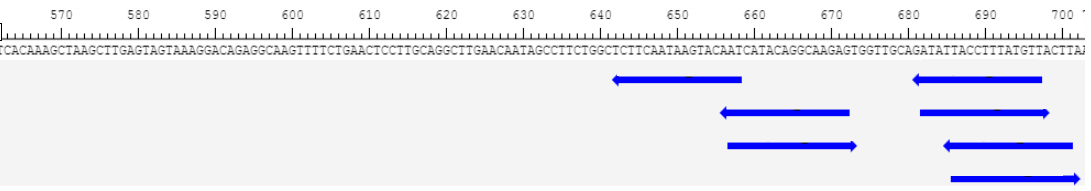
*

*
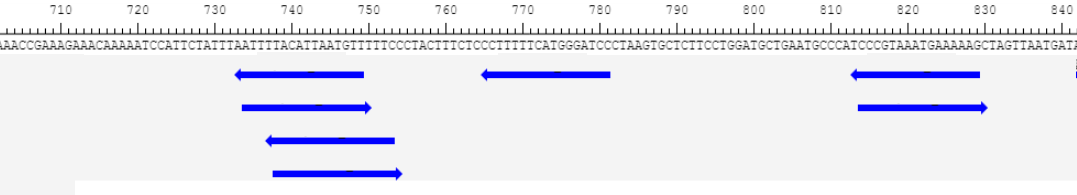
*

*
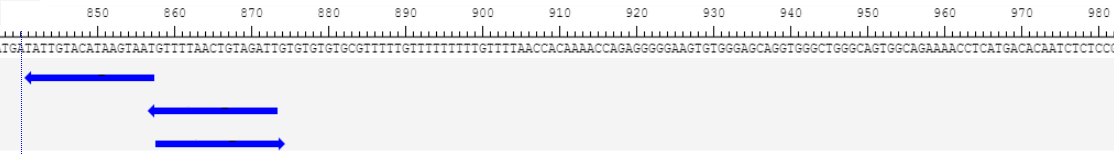
*

*
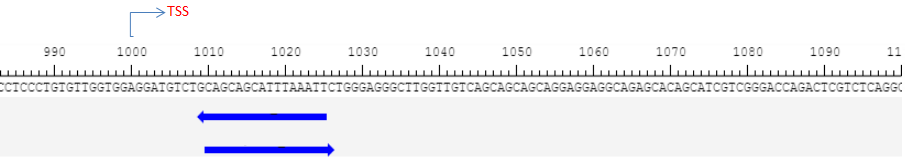
*
